# Efficacy, effectiveness, and safety of herpes zoster vaccine in the immunocompetent and immunocompromised subjects: A systematic review and network meta-analysis

**DOI:** 10.3389/fimmu.2022.978203

**Published:** 2022-09-30

**Authors:** Yue Xia, Xue Zhang, Liuren Zhang, Chuanxi Fu

**Affiliations:** Institute of Infectious Disease and Vaccine, School of Public Health, Zhejiang Chinese Medical University, Hangzhou, China

**Keywords:** immunocompetent, immunocompromised, network meta-analysis, recombinant zoster vaccine, zoster vaccine live

## Abstract

**Objective:**

To investigate the efficacy, effectiveness and safety of recombinant zoster vaccine (RZV) and zoster vaccine live (ZVL) in immunocompetent and immunocompromised subjects.

**Methods:**

Data sources: PubMed, EMBASE, Cochrane Library, and Web of Science databases (up to Jan 2022) were searched to identify English articles. Search terms included randomized controlled trials (RCTs), observational studies, herpes zoster, RZV, ZVL. Study Selection: Only randomized controlled trials (RCTs) evaluating vaccine efficacy and safety and observational studies assessing vaccine effectiveness (after a vaccine was approved for marketing) were included. Data Extraction: Two researchers independently screened the literature, extracted the data, and checked the each other results.

**Results:**

Seventeen RCTs and 19 cohort studies were included. Among immunocompetent subjects, RZV was superior to ZVL at wide intervals (relative vaccine efficacy: 84%, 95% CI: 53%–95%; relative vaccine effectiveness: 49%, 95% CI: 21%–67%), across genders and subjects aged ≥ 60 years. Among immunocompromised subjects, RZV was superior to placebo in terms of vaccine efficacy (60%, 95% CI: 49%–69%). There was no difference between ZVL and placebo in those with selected immunosuppressive conditions. RZV was 45% (95% CI: 30%–59%) superior to ZVL in real-world practice. Compared with placebo, adverse events related to RZV were primarily related to injection-site and systemic, and RZV did not increase the risk of serious adverse events (SAEs) or death. There was no difference in the incidence of adverse events between groups with and without immunosuppression.

**Conclusions:**

Both RZV and ZVL can reduce the risk of herpes zoster in both immunocompetent and immunocompromised subjects. RZV was well-tolerated in the study population and demonstrated stronger protection than ZVL.

**Systematic review registration:**

Prospero CRD42022310495.

## 1 Introduction

Herpes zoster (HZ/shingles) is an infectious disease caused by reactivation of the varicella-zoster virus (VZV) latent in the dorsal or cranial ganglion (1). HZ manifests as a band-like vesicular rash with severe and disabling pain on one side of the body (2). The most common complication of HZ is post-herpetic neuralgia (PHN), which persists for >3 months after rash onset. HZ can be complicated by ocular and visceral disorders (3).

HZ is a global public health burden, and approximately 20–30% of individuals develop HZ in their lifetime (4). According to a systematic review, the incidence rate (IR) of HZ was 5.23–10.9/1,000 person-years (PY) in Europe and 6.6–9.03/1, 000 PY in North America (5). Due to impaired cell-mediated immunity, ageing and immunocompromised conditions are the most common factors associated with increased risk of HZ (6, 7). The overall incidence of HZ in those aged >50 years was 6.4/1,000 PY (95% confidence interval (CI): 6.44–6.84), with 8.58 (95% CI: 7.72–9.51) for the age group of 70–74 and 15.94 (95% CI: 14.77–17.17) in immunocompromised subjects in China (8). In England and Germany, the HZ incidence in immunocompromised subjects was found to be 1.25–1.75 times than that of immunocompetent subjects (9, 10). In Israel, the incidence of HZ was 12.80/1,000 PY in immunocompromised subjects compared to 3.46/1, 000 PY in the general population (11, 11). In one study which had estimated the incidence of HZ in adults with immunosuppression of various severities, a nearly 40% higher incidence of HZ was observed in highly immunocompromised (HIV-positive, stem cell transplantation recipients (SCT), and organ transplantation (OT) recipients) patients than in those with low severity, such as autoimmune diseases (AID) and systemic use of corticosteroids (12, 13). HZ complications and hospitalizations are more common among immunocompromised patients (10%) than in immunocompetent patients (4.2%) (13).

Vaccination is an effective prophylactic measure to reduce the burden of disease caused by HZ. The live zoster vaccine (ZVL, ZOSTAVAX; Merck) is a live attenuated vaccine licensed in 2006 for preventing HZ in those aged ≥ 60 years. The scope of this vaccine was then extended to adults aged ≥50 years in 2011 by the Food and Drug Administration (FDA) (14, 15). However, the efficacy of ZVL may decline with increasing age, and it is generally contraindicated in immunocompromised populations because of its potential infection hazards (16, 17). The recombinant zoster vaccine (RZV, SHINGRIX; GlaxoSmithKline) is a subunit vaccine containing an adjuvant recommended for use in adults aged ≥ 50 years in 2017 by the Advisory Committee on Immunization Practices (ACIP) (18). The efficacy of RZV is high, even in those aged ≥ 70 years (19). Pooled analyzes showed that vaccine efficacy was 91.3% against HZ in participants aged 70+ years (20, 21). RZV also demonstrated 68.2% efficacy against HZ in autologous hematopoietic stem cell transplant (auto-HSCT) recipients and 87.2% efficacy in patients with hematologic malignancies in *post-hoc* efficacy analyzes (22, 23). In October 2021, ACIP approved RZV for preventing HZ in adults aged 19+ years who are or will be at an increased risk of HZ due to immunodeficiency or immunosuppression caused by known diseases or therapy (24).

Besides vaccine efficacy reported in clinical trials, post-licensed vaccine effectiveness is usually evaluated in clinical practice under real-world conditions from a public health perspective (25). A cohort study reported that ZVL could reduce HZ risk by 55% in immunocompetent subjects aged ≥ 60 years (26). The present evidence shows that the effectiveness of ZVL was 48% among general population while 37% among immunosuppressed individuals (27). Immunization with RZV reduces the risk of HZ by 85.5% in immunocompetent individuals (28). In the general medicare population, RZV effectiveness in preventing HZ was 70.1% and was effective in 64.1% of the immunocompromised beneficiaries (29).

Clinical trials and real-world studies have investigated the efficacy, effectiveness, and safety of HZ vaccines. Although ZVL is contraindicated in the immunocompromised candidates, individuals with low severity of immunosuppression (i.e., autoimmune diseases and end-stage renal diseases) receive the vaccine in clinical trials as well as in general practice. It is necessary to assess vaccine performance among individuals who are particularly at a high risk of developing HZ. In addition, the safety assessment of RZV in immunocompromised subjects is important because of the heterogeneity of herpes zoster risk within and across immunocompromised groups, the novel adjuvant and noted reactogenicity of the vaccine (24). Since there have been no reported head-to-head design studies comparing the two vaccines directly, network meta-analysis, an extension of pairwise meta-analysis, can help perform an indirect comparison through the same control (30). Therefore, we performed this systematic review and network meta-analysis to compare the efficacy, effectiveness, and safety of RZV and ZVL in the immunocompetent and immunocompromised subjects.

## 2 Methods

### 2.1 Protocol

A protocol was prepared in accordance with the Cochrane Handbook and Preferred Reporting Items for Systematic Reviews and Meta-analysis for Protocols (PRISMA-P) (31, 32). The final version of the protocol was registered in PROSPERO (CRD42022310495).

### 2.2 Eligibility criteria

PICOS (population, intervention, comparator, outcome and study) was used to determine eligibility criteria. The study population consisted of healthy as well as immunocompromised adults. Immunocompromised diseases include solid organ cancer, hematologic system cancer, solid OT, hematopoietic stem cell transplantation, human immunodeficiency virus (HIV) infection/acquired immune deficiency syndrome (AIDS), end-stage renal disease, congenital immune deficiency, and autoimmune diseases (rheumatoid arthritis, systemic lupus erythematosus, inflammatory bowel disease, multiple sclerosis, rheumatoid polymyalgia, psoriasis, autoimmune thyroiditis, type I diabetes, vasculitis and other autoimmune or collagenous connective tissue diseases) (**Appendix S1**) (33).

All participants received RZV. Comparator(s) were ZVL or placebo/unvaccinated. Experimental HZ vaccines were excluded. The efficacy, effectiveness, and safety of RZV and ZVL were compared between immunocompetent and immunocompromised subjects. The primary outcomes were vaccine efficacy and vaccine effectiveness in preventing HZ onset. Secondary outcomes were a) vaccine efficacy prevention of post-herpetic neuralgia; b) effectiveness in preventing PHN and HZO; and c) vaccine safety, including adverse events at the injection site (redness, swelling, pain), systemic adverse events (SAEs) (fatigue, myalgia, headache, gastrointestinal), as well as SAEs and death due to HZ vaccination.

Only randomized controlled trials (RCTs) evaluating vaccine efficacy and safety were included. We selected only reports in the English language. Studies reporting different dosages, potencies, and routes of administration of the same vaccine were excluded. Studies wherein the intervention measure in the control group was not placebo or included other vaccines were also excluded. The evaluation of ZVL was evaluated in subjects with immunosuppression of low severity.

Studies selected to assess vaccine effectiveness included real-world observational studies (after a vaccine was approved for marketing) without restriction on whether it was a prospective or retrospective design.

The publication time and follow-up period of the included studies were eligible, and both published and unpublished papers were qualified. In the case of different reports from the same cohort, we selected those which met expectations.

### 2.3 Information sources and literature search

Two researchers (XZ and YX) independently screened the literature, extracted the data, and checked the each other results. Databases (Web of Science, PubMed, Embase, and Cochrane Library) were screened from the founding date to 31 January, 2022. Disagreements were resolved by discussion until a consensus was reached or determined by the third member of the study team (LRZ), who then reviewed the search strategy using the PRISMA 2020 Checklist. The included studies were imported into Endnote by XZ, and duplicate studies were eliminated. A supplementary search of the grey literature (i.e., studies that are difficult to locate and unpublished studies) was conducted on study registry websites (e.g., ClinicalTrials.gov), grey literature databases (e.g., SIGLE), conference abstracts, and dissertations (**Appendix S3**).

### 2.4 Data items and abstraction process

The irrelevant studies were excluded by reading titles, furthermore, the abstracts and full texts of remain studies were reviewed to include eligible studies into our following analysis. E-mails and telephones were used to contact the original authors to obtain uncertain but important information. Extracted data included basic information (title, name of first author, publication time, area, etc.), demographic data of study subjects (sample size, gender, age, etc.), intervention measures (type of vaccine, immunization schedule, dosages, etc.), key elements of bias risk assessment, as well as outcome index and data. A draft data collection form was created after consulting with the research team.

### 2.5 Risk of bias assessment

Each study included was assessed for internal (amount of selection, information, and confounding bias) and external (generalizability of study results) validity using the Cochrane Risk of Bias Tool, which has been tested for internal consistency and reliability, and validity (31).

### 2.6 Statistical analysis

Pairwise and network meta-analyzes (NMA) were performed. The Mantel–Haenszel (MH) method was used to compare the efficacy and effectiveness of RZV and ZVL among immunocompetent and immunocompromised participants (34). When the assumption of methodological and clinical homogeneity is justified, the MH-NMA method is reliable for analyzing binary variables. Relative risk values were used to compare the efficacies of RZV and ZVL. In the efficacy meta-analysis, we performed an age subgroup analysis to obtain more reliable results. Vaccine efficacy was calculated as:


Vaccine efficacy=[(1−RR)×100]%

In the effectiveness meta-analysis, incidence density was calculated using PY and number of cases, and network meta-analysis was performed for age, sex, immunocompromised subjects, and complication subgroups, represented in the results as the incidence rate ratio (IRR). Vaccine effectiveness was calculated as:


Vaccine effectiveness=[(1−IRR)×100]%

To ensure that the transitivity assumption was upheld, we plotted the central tendencies (e.g., means and medians) of the study and subjects’ characteristics for each treatment comparison to allow for visual inspection. Examining the consistency assumption was not possible since no closed loops were included in the networks (35–38). To account for anticipated methodological and clinical heterogeneity across studies and to achieve the highest generalizability in the pooled treatment effects, we applied a random-effects model in NMA. Relative vaccine efficacy/effectiveness was used to report the protection of RZV compared to that of ZVL.

We used pairwise meta-analysis to analyze the safety of vaccines because there is no general classification of adverse events. A P value>0.05 in the heterogeneity test was considered to indicate no heterogeneity, and sensitivity analysis was used to exclude studies with low quality. We used a random-effects model when I2≥40%; otherwise, a fixed-effects model was employed. The RR and 95% CI were calculated to estimate the safety of the two vaccines. Sensitivity analysis was performed to estimate the stability of the results, and Egger’s test statistics was used to assess publication bias. The subjects of interest were divided into immunocompetent or immunocompromised group. Subgroup analysis was performed in R version 4.1.0 (R Foundation for Statistical Computing, Vienna, Austria) to compare the efficacy, effectiveness, and safety of the two vaccines in the two groups.

## 3 Results

### 3.1 Literature search

**Figure 1:**
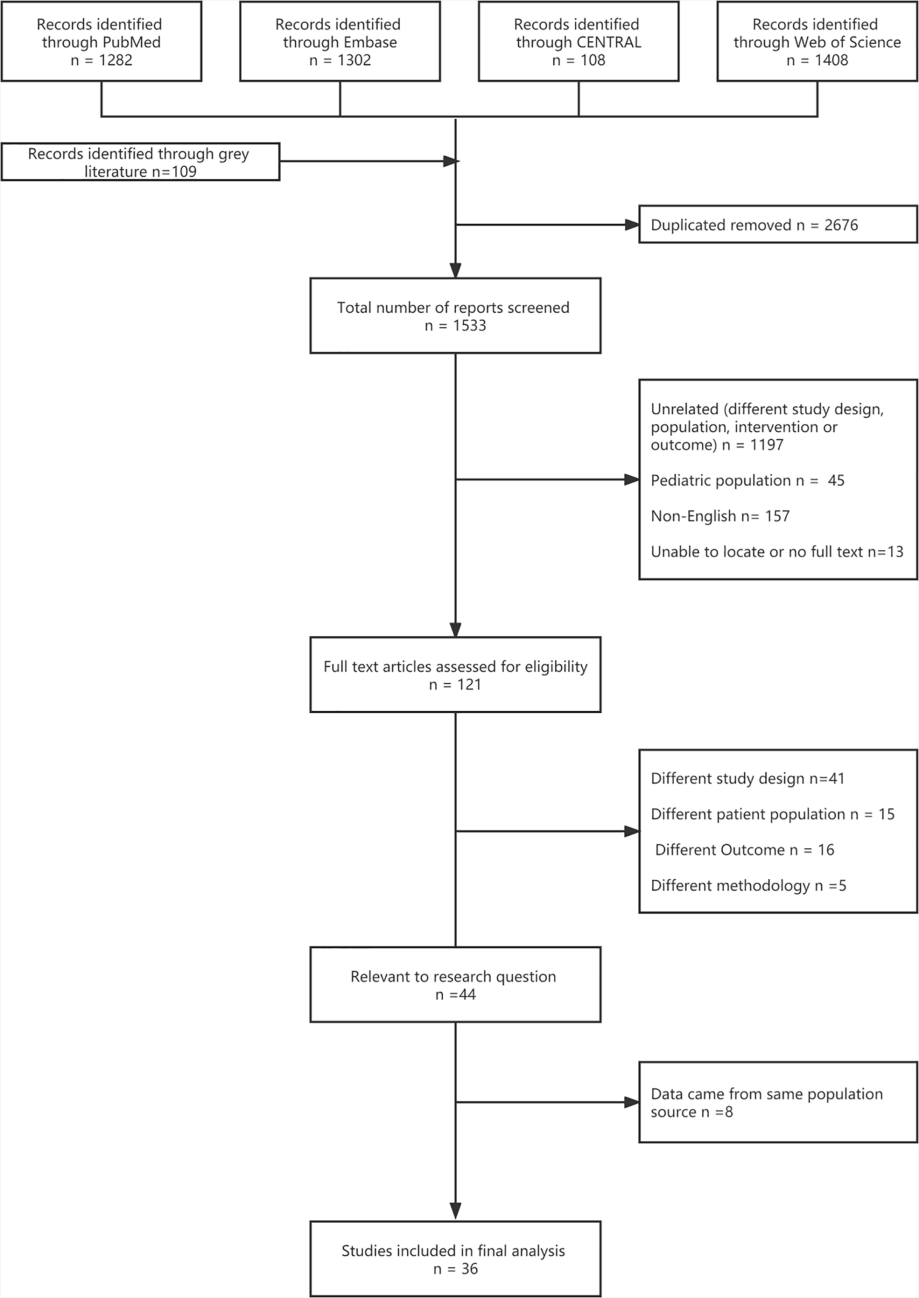
Study flow diagram.

Seventeen RCTs (16, 17, 20–23, 39–49) and 19 cohort studies (26, 27, 50–59) were finally included in the analysis after screening 1,533 titles and abstracts and 121 full text articles (**Figure 1**, **Appendix S2**). The bias risk assessment of the studies is presented in **Appendix S4–6**.

### 3.2 Study and subjects’ characteristics

Thirty-six studies were published between 2005 and 2021: 18 in in North America (50%), 4 in Europe (11%), 1 in Asia (3%), 1 in Oceania (3%), and 12 across geographical regions (33%) (**Table 1**). The median study duration was 41.5 months. In the efficacy and safety analyzes, ten studies (59%) included immunocompromised subjects. Fourteen studies (82%) included subjects with no history of HZ (**Table 1**). In the effectiveness analysis, four studies included subjects vaccinated with RZV, and 15 studies included subjects who were vaccinated with ZVL (**Table 1**). Although the case-control design is commonly used to evaluate vaccine effectiveness, case-control studies were excluded from the exclusion criteria.

**Table 1:** Characteristics of included articles.

First Author, Year	Geographical region	Study setting/design	Study Period	Health status	% Female	Patient age	Trial arms
**RZV**							
Edward A. Stadtmauer, 2014 (49)	the United States	Multi-center/RCT	2 years	autologous HCT	35.0	59.0 (20–70)	2 doses gE/AS01B, 3 doses gE/AS01B or gE/AS01E, or saline
Elchonon M. Berkowitz, 2015 (46)	Multi-continent	Multi-center/RCT	2 years	HIV-infected	5.7	46.0 (23–74)	3 doses of RZV or saline
Adriana Bastidas, 2019 (22)	Multi-continent	Multi-center/RCT	4 years	autologous HSCT	37.1	54.8 (18–78)	RZV or placebo
Alemnew F Dagnew, 2019 (23)	Multi-continent	Multi-center/RCT	2 years	haematological malignancies	40.3	56.8±15.5	RZV or placebo
Peter Vink, 2019 (47)	Multi-continent	Multi-center/RCT	3 years	solid tumors	59.8	57.1±10.8	RZV or placebo
Peter Vink, 2020 (48)	Multi-continent	Multi-center/RCT	3 years	renal transplant	28.8	52.3±12.5	RZV or placebo
Roman Chlibek, 2013 (40)	Multi-continent	Multi-center/RCT	0.5 years	immunocompetent	54.0	65.0 ± 8.9	gE combined with a adjuvant, unadjuvanted gE, or saline
A.L. Cunningham, 2016 (21)	Multi-continent	Multi-center/RCT	5 years	immunocompetent	54.9	75.6 (62–96)	RZV or placebo
Himal Lal,2015 (20)	Multi-continent	Multi-center/RCT	1 years	immunocompetent	61.2	62.3±9.0	RZV or placebo
Izurieta, H. S.2021 (29)	the United States	prospective cohort	2 years	immunocompetent and immunocompromised	NA	aged ≥65 years	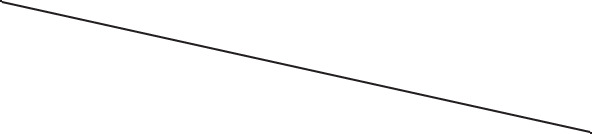
Lu, A.2021 (59)	the United States	retrospective cohort	2 years	immunocompetent	47.8	65 (56–73)	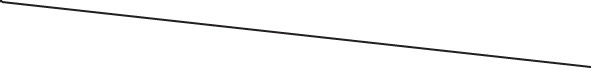
Sun, Y.2021 (60)	Hawaii (The United States)	retrospective cohort	2 years	immunocompetent	51.5	61 (54–69)	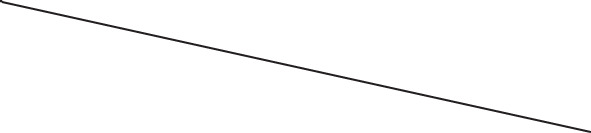
Sun, Y.2021 (28)	the United States	retrospective cohort	2 years	immunocompetent	52.2	65 (56–73)	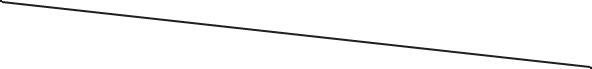
**ZVL**							
Amy F. Russell, 2015 (42)	Multi-continent	Multi-center/RCT	3 years	maintenance systemic corticosteroid therapy	71.2	69.8 (60–88)	ZVL or placebo
Constance A. Benson,2018 (43)	the United States	Multi-center/RCT	2 years	HIV on ART	16.0	49 (44–56)	ZVL or placebo
Chi ChiuMok,2019 (45)	Hong Kong (China)	Single center/RCT	1 years	SLE	93.3	45.9±15.4	ZVL or placebo
Miller, G.,2018 (44)	the United States	Multi-center/RCT	4 years	ESRD	24.0	51.9 (26–72)	ZVL or placebo
M.N. Oxman,2005 (17)	the United States	Multi-center/RCT	4 years	immunocompetent	41.0	69.0	ZVL or placebo
alexander V. Murray, 2011 (39)	Multi-continent	Multi-center/RCT	2 years	immunocompetent	58.8	70.5±7.5	ZVL or placebo
Kenneth E. Schmader, 2012 (16)	Multi-continent	multicenter/RCT	2 years	immunocompetent	61.9	54.9±2.8	ZVL or placebo
Joost N. Vermeulen, 2012 (41)	Multi-continent	multicenter/RCT	2 years	immunocompetent	63.2	68.7±7	ZVL or placebo
Tseng, H. F.2011 (26)	Southern California (the United States)	retrospective cohort	3 years	immunocompetent	53.2	69.6 (6.8)	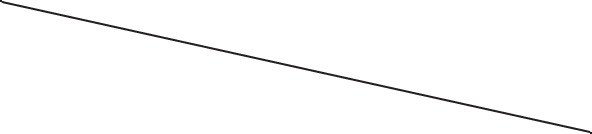
Zhang, J.2011 (61)	the United States	retrospective cohort	4.7 years	inflammatory and autoimmune diseases	62.2	aged ≥50 years	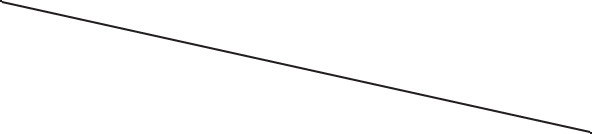
Langan, S. M.2013 (27)	the United States	retrospective cohort	3 years	immunocompetent and immunocompromised	67.6	aged ≥65 years	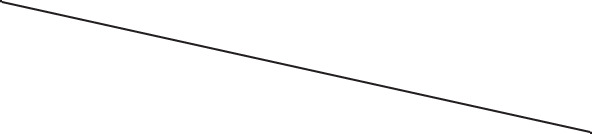
Tseng, H. F.2014 (62)	Southern California (the United States)	retrospective cohort	6 years	chemotherapy with myelosuppressive agents	58.4	74.72 (7.9)	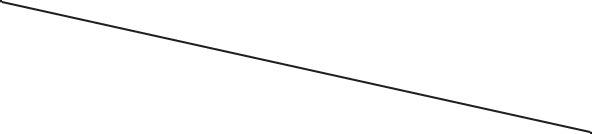
Tseng, H. F.2016 (52)	Southern California (the United States)	retrospective cohort	8 years	immunocompetent	53.6	68.7 (7.7)	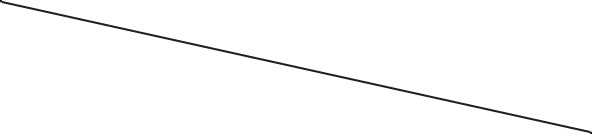
Tseng, H. F.2016 (55)	Southern California (the United States)	retrospective cohort	7 years	end-stage renal-disease	42.8	68.4 (8.9)	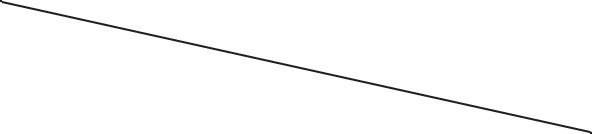
Izurieta, H. S.2017 (53)	the United States	retrospective cohort	7.5 years	immunocompetent	67.0	77.0 (6.2)	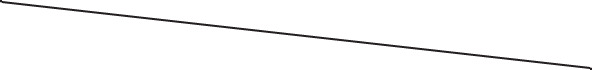
Matthews, I.2018 (50)	the United Kingdom	retrospective cohort	2.7 years	immunocompetent	52.6	aged 70–79 years	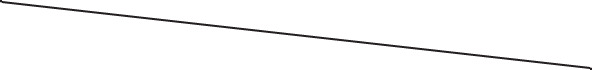
Walker, J. L.2018 (54)	the United Kingdom	retrospective cohort	3 years	immunocompetent	NA	aged 68–70 and 76–79 years	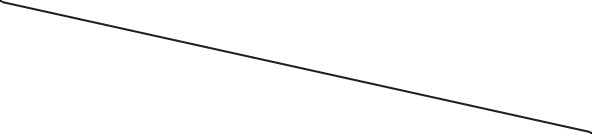
Blom, K.2019 (56)	Stockholm (Sweden)	retrospective cohort	1 years	immunocompetent	66.8	aged ≥50 years	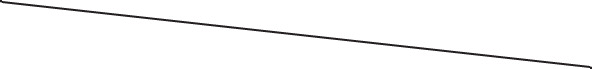
Klein, N. P.2019 (63)	Northern California (the United States)	prospective cohort	10 years	immunocompetent and immunocompromised	NA	aged ≥50 years	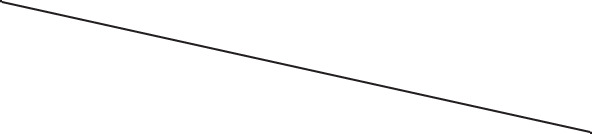
Lin, J.2021 (57)	Australia	retrospective cohort	2 years	immunocompetent	51.7	73.9 (2.8)	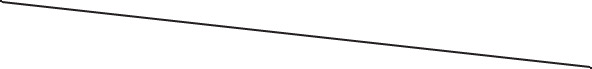
Yun, H.2017 (58)	the United States	retrospective cohort	8 years	autoimmune diseases	69.7	73.5 (7.3)	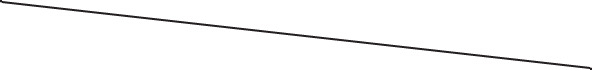
Bollaerts, K.2019 (64)	the United Kingdom	retrospective cohort	4 years	immunocompetent and immunocompromised	53.0	aged ≥70 years	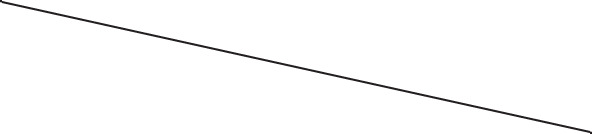
Zhang, J.2012 (51)	the United States	retrospective cohort	4 years	Immune-mediated diseases	72.3	74.0 (8.0)	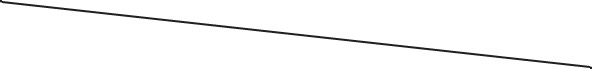

### 3.3 Sensitivity analysis

The transitivity plots showed that when two vaccines and placebo were reported, the effect modifiers were balanced between different treatments (**Appendix S7**). In the sensitivity analysis, studies with high heterogeneity were excluded by the funnel plots to obtain reliable results (**Appendix S8**). There was no publication bias in any of the literature included in this study (**Appendix S8**).

### 3.4 Vaccine efficacy

#### 3.4.1 Immunocompetent subjects

**Figure 2:**
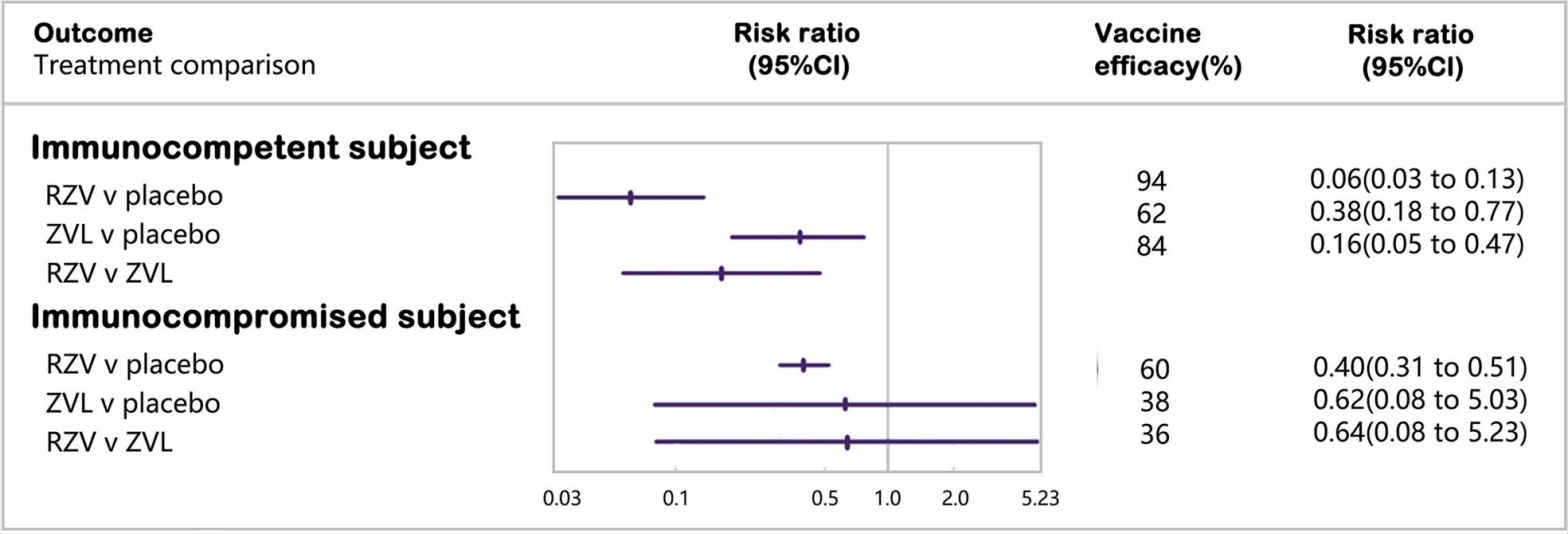
Forest plot of vaccine efficacy in reducing cases of herpes zoster.

Four RCTs enrolling 80,980 immunocompetent subjects were included in the network meta-analysis of laboratory or clinically confirmed cases of HZ (16, 17, 20, 21) (**Figure 2**). RZV was statistically superior to placebo (vaccine efficacy: 94%, 95%CI: 87%–97%) and ZVL (relative vaccine efficacy: 84%, 95% CI: 53%–95%). ZVL was statistically superior to the placebo (vaccine efficacy: 62%, 95% CI: 23%–82%).

We performed age subgroup analysis (50–59, 60–69, 70+ years) to analyze RZV efficacy and found no statistical difference among the subgroups. No age-specific information was found in the ZVL studies that supported our age subgroup analysis (**Appendix S9**).

#### 3.4.2 Immunocompromised subjects

Six RCTs including 3,284 immunocompromised subjects with laboratory or clinically confirmed HZ, were included in the network meta-analysis (22, 23, 42, 43, 46, 49). RZV was statistically superior to placebo (vaccine efficacy: 60%, 95% CI: 49%–69%), while ZVL was not statistically different from placebo or RZV in subjects with selective immunosuppression with low severity (**Appendix S9**).

#### 3.4.3 Prevention of PHN

The number of studies on the efficacy of the two vaccines to prevent PHN are very limited, with only three, so we were unable to conduct a meta-analysis and only reported their findings. Studies have found that in immunocompetent subjects, the efficacy of RZV against PHN in subjects over 50 years old was 91.2% (95%CI: 75.9%-97.7%), and the efficacy in subjects over 70 years old was 88.8% (95%CI: 68.7% - 97.1%). As shown in one study, the curative effect of ZVL was 66.5% (95% CI: 47.5% 79.2%) (17, 21). Only one study reported an 89% (95% CI: 22%–100%) efficacy of RZV against PHN in patients with HSCT and an 85% (95% CI: 32%–97%) efficacy against hospitalizations (22).

### 3.5 Vaccine effectiveness

#### 3.5.1 Immunocompetent subjects

**Figure 3:**
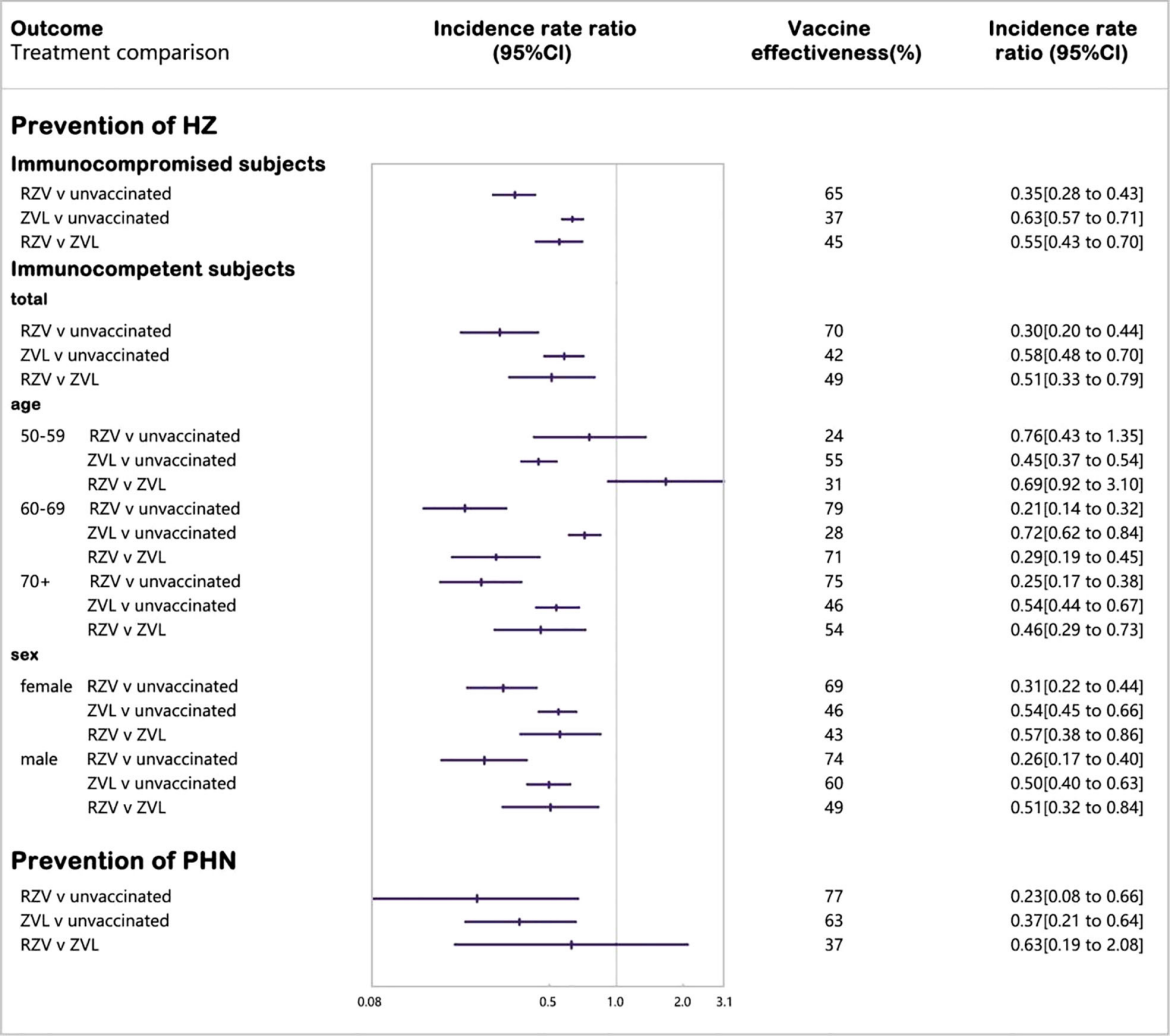
Forest plot of vaccine effectiveness in reducing cases of herpes zoster.

Nine cohort studies were included in the network meta-analysis of laboratory or clinically confirmed cases of HZ (26, 28, 50, 52–54, 57, 60, 64) (**Figure 3**). Vaccination with RZV was statistically superior to no vaccination (vaccine effectiveness: 70%, 95% CI: 56%–80%) and ZVL (relative vaccine effectiveness: 49%, 95% CI: 21%–67%). Vaccination with ZVL was statistically superior to no vaccination (vaccine effectiveness: 42%, 95% CI: 30%–52%).

We divided the immunocompetent subjects based on age and sex. We included four studies in the 50–59 age group, six studies in the 60–69 age group, and eight studies in the 70+ age group (26, 28, 50, 52–54, 56, 57, 60, 62, 65). Seven studies were included in each sex subgroup (26, 28, 52–54, 57, 60).

To assess age-related vaccine effectiveness, we divided the subjects based of the following three age groups: 50–59, 60–69, and 70+. RZV was significantly more protective than ZVL in subjects aged 60+ years (60–69 years old, relative vaccine effectiveness: 71%; 70+ years old, relative vaccine effectiveness: 54%), and the protective effect of RZV decreased with an increase in age.

By analyzing sex, we found that both vaccines were less protective in females than in males, and RZV was statistically more protective than ZVL in females (relative vaccine effectiveness: 43%) and male subjects (relative vaccine effectiveness: 49%).

#### 3.5.2 Immunocompromised subjects

Eight cohort studies were included in the network meta-analysis of laboratory or clinically confirmed cases of HZ (27, 29, 51, 55, 58, 61, 62, 64) (**Figure 3**). Vaccination with RZV was statistically superior to no vaccination (vaccine effectiveness: 65%, 95% CI: 57%–72%) and ZVL (relative vaccine effectiveness: 45%, 95% CI: 30%–59%). Vaccination with ZVL in subjects with selective immunosuppression of low severity was statistically superior to no vaccination (vaccine effectiveness: 37%, 95% CI: 29%–43%).

We analyzed the effectiveness of ZVL by age group (**Appendix S10**). We found that vaccination with RZV was statistically superior to no vaccination (vaccine effectiveness: 36%, 95% CI: 26%–45%). The age-related effectiveness of the ZVL was not statistically significant in those aged 60+ years. The age-related effectiveness of RZV has not been assessed in relevant studies.

#### 3.5.3 Prevention of PHN

Five cohort studies were included in the network meta-analysis of laboratory or clinically confirmed cases of PHN (27, 29, 50, 54, 63). Vaccination with R-1ZV was statistically superior to no vaccination (vaccine effectiveness: 77%, 95% CI: 34%–92%). Vaccination with ZVL was considered statistically superior to no vaccination (vaccine effectiveness: 63%, 95% CI: 36%–79%), while ZVL was not statistically different from RZV.

#### 3.5.4 Prevention of HZO

We included three studies in the pairwise meta-analysis of HZO (**Appendix S11**). Vaccination with RZV was statistically higher than no vaccination (vaccine effectiveness: 67%, 95% CI: 62%–71%). Vaccination with ZVL to prevent HZO has not been assessed in relevant studies.

### 3.6 Vaccine safety

#### 3.6.1 RZV

##### 3.6.1.1 Injection sites

We performed a pairwise meta-analysis to identify whether subjects vaccinated with RZV had a higher rate of redness (RR: 30.09, 95% CI: 23.95–37.81), swelling (RR: 24.89, 95% CI: 19.25–32.17) and pain (RR: 7.79, 95% CI: 6.66–9.11) than those receiving placebo. The risk of RZV at the injection site was not statistically significant between immunocompetent and immunocompromised subjects (**Appendix S12**).

##### 3.6.1.2 Systemic adverse events

Higher reported incidence of fatigue (RR: 2.26, 95% CI: 1.88–2.73), myalgia (RR:4.01, 95% CI: 3.11–5.17), headache (RR: 2.43, 95% CI: 2.26–2.61), and gastrointestinal symptoms (RR: 1.29, 95% CI: 1.13–1.47) were noted in the RZV group than in the placebo group, which was not statistically significant between the immunocompetent and immunocompromised groups (**Appendix S12**).

##### 3.6.1.3 Serious adverse events and death

There was no statistical difference between RZV and placebo with regard to either the reported serious adverse events (RR: 0.97, 95% CI: 0.92–1.03) or death (RR: 0.93, 95% CI: 0.85–1.03), wherein no difference was found between the immunocompetent and immunocompromised subjects (**Appendix S12**).

#### 3.6.2 ZVL

##### 3.6.2.1 Injection sites

Compared with the placebo, there was no extra risk of adverse events at the injection sites after receiving ZVL (RR: 2.91, 95% CI: 2.68–3.16), and no difference was noted between immunocompetent and immunocompromised subjects (**Appendix S13**).

##### 3.6.2.2 Systemic adverse events

A higher incidence of systemic adverse events was noted in the ZVL group than in the placebo group (RR: 1.06, 95% CI: 1.02–1.09), and the risk was similar between immunocompetent and immunocompromised subjects (**Appendix S13**).

##### 3.6.2.3 Serious adverse events and death

There was a higher incidence of severe adverse events in the ZVL group than in the placebo group (RR: 1.18, 95% CI: 1.04–1.34); however, a difference in the risk of death was not noted for ZVL (RR: 0.99, 95% CI: 0.90–1.09). Serious adverse events and death due to ZVL were similar between immunocompetent and immunocompromised subjects (**Appendix S13**).

## 4 Discussion

In this systematic review and meta-analysis, we compared HZ vaccines (RZV and ZVL) in two groups (immunocompetent and immunocompromised subjects) in two scenarios (clinical trials and real-world practice). These findings suggest that RZV is superior to ZVL in reducing the risk of developing HZ in both immunocompetent and immunocompromised subjects. RZV is considered to be generally safe, while ZVL might slightly increase the risk of SAEs.

Both the vaccines can offer protection against developing HZ among immunocompetent subjects, and RZV shows better performance than ZVL with a relative efficacy of 84% and relative effectiveness of 49%. RZV efficacy is robust across age groups in the subgroup analysis indicating the strong immune response provided by adjuvant subunit antigens (66). In contrast, ZVL efficacy declines with increasing age, representing the more robust VZV-specific cell-medicated immunity among the younger subjects than the elderly. As age is a strong predictor when evaluating the protection of vaccine-induced immune response, our results reconfirm the protective effect and general applicability of RZV. The vaccines similarly reduce the risk of HZ in either sex. Although female is an independent risk factor for HZ, vaccination can also protect these subjects from contracting HZ (67). Since antiviral treatment is recommended to be given to HZ patients within 72 hours after the rash onset and the role in preventing PHN is less clear, vaccination can reduce the disease burden and prevent HZ related complications.

Among the immunocompromised subjects who are vulnerable to infectious diseases and can develop serious complications, vaccination may be less protective owing to impaired immune function (65). RZV is found to provide an extra 36% protection in RCTs and 45% protection in medical practice compared with ZVL. ZVL efficacy estimate is not statistically significant, probably due to the limited number of reported positive cases. Data on ZVL vaccination in immunocompromised subjects are limited, which is in part due to the recommendation for use of live vaccine in these individuals is inexplicit. Additionally, ZVL was evaluated in subjects with low severity of selective immunosuppression. RZV is demonstrated higher protection effect in real-world practice. Potential explanations for this disparity include differences in the composition of study subjects and subjects with various degrees of immunosuppression. In practice, individuals with severe immunosuppression are not usually recommended to receive RZV, resulting in higher vaccine effectiveness. Nevertheless, since RZV was approved for use in immunocompromised subjects in 2021, further studies are needed to confirm its effectiveness.

The efficacy and effectiveness of RZV in the immunocompromised subjects are lower than those in immunocompetent subjects, reflecting cell-mediated immunodeficiency and a weaker immune response due to underlying immunocompromised status (22). RZV confers greater protection against HZ than ZVL, which is attributed to glycoprotein E (gE) adjuvanted with AS01_B_, which can enhance VZV-specific T-cell memory immune responses (20). A clinical trial comparing T-cell memory responses to the two vaccines found higher responses in RZV recipients, and only RZV recipients had a five-year persistence of higher responses (68, 69). Since the increased risk of HZ is associated with female sex, ageing, and immunocompromised condition, vaccination of these groups should be encouraged proactively (11).

For safety outcomes, our subgroup analysis showed no difference between the immunocompetent and immunocompromised groups, which indicated that immunosuppression might not be a determinant of vaccine adverse reactions. RZV recipients developed more injection-site and systemic adverse events than placebo recipients, indicating that the adjuvant can increase overall immune responses to antigen. The reactions are generally mild-to-moderate, and RZV does not increase the risk of SAEs or death. Compared with placebo, the incidence of SAEs in the ZVL is slightly higher. SAEs in the ZVL group were only reported in the SPS study, which showed no clinically difference (17).

In conclusion, our study highlights the protection provided by the HZ vaccine against HZ and associated complications. RZV vaccine protected immunocompromised subjects against HZ infection without safety concerns. ZVL is less effective than RZV and has a limited utility in recipients with immunosuppression. The results of this systematic review and network meta-analysis are helpful when considering recommendations for the use of HZ vaccines in elderly adults and immunocompromised subjects.

## 5 Strengths and limitations

Our study has several strengths. First, since there are currently no studies that directly compare the efficacy, effectiveness and safety of two herpes zoster vaccines in the immunocompetent and immunocompromised subjects, our study indirectly compares the two vaccines through rigorous and effective meta-analysis, providing evidence on recommendation of HZ vaccination for the clinicians and policy makers. Second, our design excludes studies in which the control group was not placebo and studies in which other vaccines were administered simultaneously, making our findings more reliable. Third, besides efficacy studies, we included real world studies to evaluate vaccine effectiveness, supporting the ACIP’s recommendation to vaccinate immunocompromised subjects with RZV.

There are limitations in our study. First, the included studies may be subject to biases, such as asymmetry in the funnel plots of HZ outcomes, which may have been caused by variations in study characteristics, resulting in a wide-ranging confidence interval. Despite this heterogeneity, our robust results suggest that most subjects at an elevated risk of developing HZ can be protected by receiving the HZ vaccine. Second, the generalizability of our review is limited by the limited number of available studies, possibly because RZV was approved only recently used for immunocompromised subjects, and ZVL is contraindicated in these groups, which precluded further analysis of vaccine performance in different types of immunocompromised conditions.

## Data availability statement

The original contributions presented in the study are included in the article/**Supplementary Material**. Further inquiries can be directed to the corresponding author.

## Author contributions

CF had full access to all of the data in the study and takes responsibility for the integrity of the data and the accuracy of the data analysis. Concept and design, YX, XZ, and CF. Acquisition, analysis, or interpretation of data: all authors. Drafting of the manuscript, YX and XZ. Critical revision of the manuscript for important intellectual content: all authors. Statistical analysis, YX. Supervision, CF and LZ. All authors contributed to the article and approved the submitted version.

## Conflict of interest

The authors declare that the research was conducted in the absence of any commercial or financial relationships that could be construed as a potential conflict of interest.

## Publisher’s note

All claims expressed in this article are solely those of the authors and do not necessarily represent those of their affiliated organizations, or those of the publisher, the editors and the reviewers. Any product that may be evaluated in this article, or claim that may be made by its manufacturer, is not guaranteed or endorsed by the publisher.
