# Supplementary material for: Efficacy, effectiveness, and safety of herpes zoster vaccine in the immunocompetent and immunocompromised subjects: A systematic review and network meta-analysis
Source: Front Immunol. 2022 Sep 30;13:978203. doi: 10.3389/fimmu.2022.978203 (PMC9561817; doi:10.3389/fimmu.2022.978203)
Supplement: Supplementary file 1 [file DataSheet_1.pdf]

Content

Appendix S1: Outcome Definitions. 2
Appendix S2: Literature Search Strategy for PubMed. 3
Appendix S3: Grey Literature Sources. 7
Appendix S4: Summary Results of the Cochrane Collaboration Risk of Bias Assessment (n=17 Randomized Controlled Trials). 8
Appendix S5: Summary Results of the Newcastle-Ottawa Scale Assessment (n=19 Cohort Studies). 9
Appendix S6: Risk of bias results. 14
Appendix S7: Three comparators were included: live attenuated vaccine, adjuvant recombinant subunit vaccine, and placebo. 15
Appendix S8: The funnel plotsand publication bias plots. 16
Appendix S9: Forest plot of RZV vaccine efficacy by age subgroup. 25
Appendix S10: Forest plot of ZVL vaccine effectiveness by age subgroup. 26
Appendix S11: Forest plot of RZV vaccine effectiveness for prevention of HZO. 27
Appendix S12: Safety outcomes of RZV. 28
Appendix S13: Safety outcomes of ZVL. 31

Appendix S1: Outcome Definitions.

Outcome	Definition
Confirmed herpes zoster infection	Subjects who underwent clinical diagnosis of herpes zoster infection through laboratory testing (e.g. polymerase-chain-reaction assay, virus culture) and/or examination by a physician were classified as having confirmed cases of herpes zoster infection.
Post-herpetic neuralgia	Subjects with pain continuing 90 days or longer after the onset of the shingles rash. All efficacy and effectiveness data are based on this definition and also known as PHN-901.
Herpes zoster ophthalmicus	Subjects with herpes zoster infection in the ocular region.
Injection site adverse events	Local reactions such as pain, redness, swelling, induration, pruritus, etc. at the injection site.
Systematic adverse events	Generalized reactions such as headache, myalgia, fatigue, etc.
Serious adverse events	Any events requiring hospitalization (initial or prolonged) or medical intervention to prevent permanent damage/impairment; resulting in birth defect, disability/permanent damage, death or life-threatening condition2.
Death	The number of subjects reported for death regardless of causality.

1 .Yawn BP. Post-Shingles Neuralgia by Any Definition Is Painful, but Is It PHN? Mayo Clinic Proceedings. 2011;86(12):1141- 1142. doi:10.4065/mcp.2011.0724.

2.US Food and Drug Administration. What is a Serious Adverse Event? [updated February 1, 206]. Available from https://www.fda.gov/Safety/MedWatch/HowToReport/ucm053087.htm [accessed January 10, 2018.

Appendix S2: Literature Search Strategy for PubMed.

Search number	Query	Search Details	Results
1	randomized controlled trial [pt]	"randomized controlled trial"[Publication Type]	564,201
2	controlled clinical trial [pt]	"controlled clinical trial"[Publication Type]	654,086
3	randomized [tiab]	"randomized"[Title/Abstract]	605,081
4	placebo [tiab]	"placebo"[Title/Abstract]	233,312
5	trial [tiab]	"trial"[Title/Abstract]	699,406
6	randomly [tiab]	"randomly"[Title/Abstract]	379,944
7	groups [tiab]	"groups"[Title/Abstract]	2,362,600
8	# 1 OR #2 OR #3 OR #4 OR #5 OR #6 OR #7		3,442,937
9	animals [mh] NOT humans [mh]	"animals"[MeSH Terms] NOT "humans"[MeSH Terms]	4,979,947
10	#8 NOT #9		2,956,001
11	herpes zoster	"herpes zoster"[MeSH Terms] OR ("herpes"[All Fields] AND "zoster"[All Fields]) OR "herpes zoster"[All Fields]	18,695
12	shingles	"herpes zoster"[MeSH Terms] OR ("herpes"[All Fields] AND "zoster"[All Fields]) OR "herpes zoster"[All Fields] OR "shingles"[All Fields] OR "shingle"[All Fields]	19, 184
13	varicella zoster virus	"herpesvirus 3, human"[MeSH Terms] OR "human herpesvirus 3"[All Fields] OR ("varicella"[All Fields] AND "zoster"[All Fields] AND "virus"[All Fields]) OR "varicella zoster virus"[All Fields]	12,306
14	zoster	"herpes zoster"[MeSH Terms] OR ("herpes"[All Fields] AND "zoster"[All Fields]) OR "herpes zoster"[All Fields] OR "zoster"[All Fields]	22,871
15	# 11 OR # 12 OR # 13 OR # 14		24,321
16	vaccine	"vaccin"[Supplementary Concept] OR "vaccin"[All Fields] OR "vaccination"[MeSH Terms] OR "vaccination"[All Fields] OR "vaccinable"[All Fields] OR "vaccinal"[All Fields] OR "vaccinate"[All Fields] OR "vaccinated"[All Fields] OR "vaccinates"[All Fields] OR "vaccinating"[All Fields] OR "vaccinations"[All Fields] OR "vaccination s"[All Fields] OR "vaccinator"[All Fields] OR "vaccinators"[All Fields] OR "vaccine s"[All Fields] OR "vaccined"[All Fields] OR "vaccines"[MeSH Terms] OR "vaccines"[All Fields] OR "vaccine"[All Fields] OR	449,444

		"vaccins"[All Fields]	
17	vaccination	"vaccin"[Supplementary Concept] OR "vaccin"[All Fields] OR "vaccination"[MeSH Terms] OR "vaccination"[All Fields] OR "vaccinable"[All Fields] OR "vaccinal"[All Fields] OR "vaccinate"[All Fields] OR "vaccinated"[All Fields] OR "vaccinates"[All Fields] OR "vaccinating"[All Fields] OR "vaccinations"[All Fields] OR "vaccination s"[All Fields] OR "vaccinator"[All Fields] OR "vaccinators"[All Fields] OR "vaccine s"[All Fields] OR "vaccined"[All Fields] OR "vaccines"[MeSH Terms] OR "vaccines"[All Fields] OR "vaccine"[All Fields] OR "vaccins"[All Fields]	449,444
18	immunization	"immune"[All Fields] OR "immuned"[All Fields] OR "immunes"[All Fields] OR "immunisation"[All Fields] OR "vaccination"[MeSH Terms] OR "vaccination"[All Fields] OR "immunization"[All Fields] OR "immunization"[MeSH Terms] OR "immunisations"[All Fields] OR "immunizations"[All Fields] OR "immunise"[All Fields] OR "immunised"[All Fields] OR "immuniser"[All Fields] OR "immunisers"[All Fields] OR "immunising"[All Fields] OR "immunities"[All Fields] OR "immunity"[MeSH Terms] OR "immunity"[All Fields] OR "immunization s"[All Fields] OR "immunize"[All Fields] OR "immunized"[All Fields] OR "immunizer"[All Fields] OR "immunizers"[All Fields] OR "immunizes"[All Fields] OR "immunizing"[All Fields]	1,424,649
19	# 16 OR # 17 OR # 18		1,567,762
20	herpes zoster vaccine	"herpes zoster vaccine"[MeSH Terms] OR ("herpes"[All Fields] AND "zoster"[All Fields] AND "vaccine"[All Fields]) OR "herpes zoster vaccine"[All Fields]	2,381
21	zoster vaccine	"herpes zoster vaccine"[MeSH Terms] OR ("herpes"[All Fields] AND "zoster"[All Fields] AND "vaccine"[All Fields]) OR "herpes zoster vaccine"[All Fields] OR ("zoster"[All Fields] AND "vaccine"[All Fields]) OR "zoster vaccine"[All Fields]	3,277
22	shingrix	"glycoprotein e varicella zoster virus"[Supplementary Concept] OR "glycoprotein e varicella zoster virus"[All Fields] OR "varicella zoster virus glycoprotein e"[All Fields] OR "shingrix"[All Fields]	274
23	zostavax	"herpes zoster vaccine"[MeSH Terms] OR ("herpes"[All Fields] AND "zoster"[All Fields] AND "vaccine"[All Fields]) OR "herpes zoster vaccine"[All Fields] OR	2,396

		"zostavax"[All Fields]	
24	#20 OR #21 OR #22 OR #23		3,415
25	# 15 AND # 19		6,834
26	#24 OR #25		6,947
27	cohort analysis	"cohort studies"[MeSH Terms] OR ("cohort"[All Fields] AND "studies"[All Fields]) OR "cohort studies"[All Fields] OR ("cohort"[All Fields] AND "analysis"[All Fields]) OR "cohort analysis"[All Fields]	2,488,563
28	longitudinal study	"longitudinal studies"[MeSH Terms] OR ("longitudinal"[All Fields] AND "studies"[All Fields]) OR "longitudinal studies"[All Fields] OR ("longitudinal"[All Fields] AND "study"[All Fields]) OR "longitudinal study"[All Fields]	305,607
29	retrospective cohort	("retrospective studies"[MeSH Terms] OR ("retrospective"[All Fields] AND "studies"[All Fields]) OR "retrospective studies"[All Fields] OR "retrospective"[All Fields] OR "retrospectively"[All Fields] OR "retrospectives"[All Fields]) AND ("cohort studies"[MeSH Terms] OR ("cohort"[All Fields] AND "studies"[All Fields]) OR "cohort studies"[All Fields] OR "cohort"[All Fields] OR "cohort s"[All Fields] OR "cohorte"[All Fields] OR "cohorts"[All Fields])	1,071, 163
30	prospective cohort	("longitudinal studies"[MeSH Terms] OR ("longitudinal"[All Fields] AND "studies"[All Fields]) OR "longitudinal studies"[All Fields] OR "prospective"[All Fields] OR "prospectively"[All Fields]) AND ("cohort studies"[MeSH Terms] OR ("cohort"[All Fields] AND "studies"[All Fields]) OR "cohort studies"[All Fields] OR "cohort"[All Fields] OR "cohort s"[All Fields] OR "cohorte"[All Fields] OR "cohorts"[All Fields])	890,209
31	cohort study	"cohort studies"[MeSH Terms] OR ("cohort"[All Fields] AND "studies"[All Fields]) OR "cohort studies"[All Fields] OR ("cohort"[All Fields] AND "study"[All Fields]) OR "cohort study"[All Fields]	2,545,673
32	#27 OR #28 OR #29 OR #30 OR #31		2,672,873
33	#26 AND #32		1,004
34	effectiveness	"effect"[All Fields] OR "effecting"[All Fields] OR "effective"[All Fields] OR "effectively"[All Fields] OR "effectiveness"[All Fields] OR "effectivenesses"[All Fields] OR "effectives"[All Fields] OR "effectivities"[All Fields] OR "effectivity"[All Fields] OR "effects"[All Fields]	10,378,764
35	#33 AND #34		416

36	("1960/01/01"[Date - Publication] : "2022/01/31"[Date - Publication])	1960/01/01:2022/01/31[Date - Publication]	32,033,207
37	#35 AND #36		413
38	# 10 AND #26		1,014
39	#38 AND #36		997
40	#37 OR #39		1282

Appendix S3: Grey Literature Sources.

1) Trial registries:

Biomed Central. ISRCTN Registry: http://www.isrctn.com/

National Institute of Medical Statistics, Indian Council of Medical Research. Clinical Trials Registry - India (CTRI): http://ctri.nic.in/Clinicaltrials/advancesearchmain.php

US National Institutes of Health. ClinicalTrials.gov: http://clinicaltrials.gov/ct/screen/AdvancedSearch

Thomson CenterWatch. CenterWatch Clinical Trials Listing Service: http://www.centerwatch.com/clinical-trials/listings/

2) General grey databases:

GreyNet International: http://www.greylit.org

SIGLE (System for Information on Grey Literature in Europe): http://www.opengrey.eu

3) International databases:

Agency for Healthcare Research and Quality: http://www.ahrq.gov/research/index.html

LILACS - Latin-American and Caribbean Center on Health Sciences Information: http://lilacs.bvsalud.org/en/

WHO (WHOLIS): http://dosei.who.int/uhtbin/cgisirsi/Tue+Apr++5+17:45:43+MEST+2016/0/49

4) Theses and dissertations:

DART-Europe E-theses Portal: http://www.dart-europe.eu/basic-search.php

Electronic Theses Online Service (ETHOS) | British Library: http://ethos.bl.uk/Home.do;jsessionid=D96E9CF245B0FE0199DDDB94FF4BD2A7

Open access dissertations: https://oatd.org

Thesis Canada Portal: http://www.bac-lac.gc.ca/eng/services/theses/Pages/theses canada.aspx

Appendix S4: Summary Results of the Cochrane Collaboration Risk of Bias Assessment (n=17 Randomized Controlled Trials).

Study ID	Random sequence generation	Allocation concealment	Blinding of participants & personnel	Blinding of outcome assessment	Incomplete outcome data	Selective reporting	Other bias
Edward A. Stadtmauer，2014	Low	Low	Low	Low	Low	Low	Unclear
Elchonon M. Berkowitz，2015	Unclear	Low	Low	Low	Low	Low	Unclear
Adriana Bastidas，2019	Low	Low	Low	Low	Low	Low	Low
Alemnew F Dagnew ，2019	Low	Low	Low	Low	Low	Low	Low
Peter Vink ，2019	Low	Low	Low	High	Low	Unclear	Unclear
Peter Vink ，2020	Low	Unclear	Low	High	Low	Low	Unclear
Roman Chlibek，2013	Low	Low	Low	Low	Low	Low	High
A.L. Cunningham,2016	Low	Low	Low	Low	Low	Low	Unclear
Himal Lal,2015	Low	Unclear	Low	Low	Low	Low	High
Amy F. Russell,2015	Low	Unclear	Low	Low	Low	Low	High
Constance A. Benson,2018	Low	Unclear	Low	Low	Low	Low	Unclear
Chi Chiu Mok, 2019	Low	Low	Low	Low	Low	Low	Low
M.N. Oxman,2005	Unclear	Unclear	Low	Low	Low	Low	High
Kenneth E. Schmader,2012	Low	Low	Low	Low	Low	Low	Unclear
Joost N. Vermeulen ，2012	Low	Low	Low	Low	Low	Low	High
Miller, G.,2018	Unclear	Low	Low	Unclear	Low	Low	Unclear
alexander V. Murray ，2011	Unclear	Low	Low	Low	Low	Low	High

Appendix S5: Summary Results of the Newcastle-Ottawa Scale Assessment (n=19 Cohort Studies).

Study		SELECTION				COMPARABILITY	OUTCOME			Total scores
		Representativeness of the Exposed Cohort	Selection of the Non-Exposed Cohort	Ascertainment of Exposure	Demonstration That Outcome of Interest Was Not Present at Start of Study	Comparability of Cohorts on the Basis of the Design or Analysis	Assessment of Outcome	Was Follow-Up Long Enough for Outcomes to Occur	Adequacy of Follow Up of Cohorts	
Recombinant Zoster Vaccine (Shingrix): Real-World Effectiveness in the First 2 Years Post-Licensure	Izurieta, H. S.2021	1	1	1	1	2	1	1	1	9
Effectiveness of the Recombinant Zoster Vaccine for Herpes Zoster Ophthalmicus in the United States	Lu, A.2021	1	1	1	1	2	1	1	1	9
Effectiveness of the recombinant zoster vaccine among Kaiser Permanente Hawaii enrollees aged 50 and older: A retrospective	Sun, Y.2021	1	1	1	1	2	1	1	1	9

cohort study										
Effectiveness of the Recombinant Zoster Vaccine in Adults Aged 50 and Older in the United States: A Claims-Based Cohort Study	Sun, Y.2021	1	1	1	1	2	1	1	1	9
Herpes Zoster Vaccine in Older Adults and the Risk of Subsequent Herpes Zoster Disease	Tseng, H. F.2011	1	1	1	1	2	1	1	1	9
The use, safety, and effectiveness of herpes zoster vaccination in individuals with inflammatory and autoimmune diseases: a longitudinal observational study	Zhang, J.2011	0	1	1	1	1	1	1	1	7
Herpes Zoster Vaccine Effectiveness against Incident Herpes Zoster and Post-herpetic Neuralgia in an Older US Population: A Cohort Study	Langan, S. M.2013	1	1	1	1	2	1	1	1	9
Vaccination Against Zoster	Tseng, H.	1	1	1	1	2	1	1	1	9

Remains Effective in Older Adults Who Later Undergo Chemotherapy	F.2014									
Declining Effectiveness of Herpes Zoster Vaccine in Adults Aged ≥60 Years	Tseng, H. F.2016	1	1	1	1	2	1	1	1	9
Effectiveness of Herpes Zoster Vaccine in Patients 60 Years and Older With End-stage Renal Disease	Tseng, H. F.2016	1	1	1	1	2	1	1	1	9
Effectiveness and Duration of Protection Provided by the Live-attenuated Herpes Zoster Vaccine in the Medicare Population Ages 65 Years and Older	Izurieta, H. S.2017	1	1	1	1	2	1	1	1	9
Assessing the effectiveness of zoster vaccine live: A retrospective cohort study using primary care data in the United Kingdom	Matthews, I.2018	1	1	1	1	2	1	1	1	9
Effectiveness of herpes zoster vaccination in an older United Kingdom population	Walker, J. L.2018	0	1	1	1	2	1	1	1	8
Effectiveness of the herpes	Blom,	0	1	1	1	2	1	1	1	8

zoster vaccine Zostavax® in Stockholm County, Sweden	K.2019									
Long-term effectiveness of zoster vaccine live for postherpetic neuralgia prevention	Klein, N. P.2019	1	1	1	1	2	1	1	1	9
Effectiveness of the live-attenuated herpes zoster vaccine 2 years after its introduction in Australia	Lin, J.2021	0	1	1	1	2	1	0	1	7
Longterm Effectiveness of Herpes Zoster Vaccine among Patients with Autoimmune and Inflammatory Diseases	Yun, H.2017	1	1	1	1	0	1	1	1	7
Risk factors for modified vaccine effectiveness of the live attenuated zoster vaccine among the elderly in England	Bollaerts, K.2019	1	1	1	1	2	1	1	1	9
Association between vaccination for herpes zoster and risk of herpes zoster infection among older patients with selected	Zhang, J.2012	1	1	1	1	2	1	1	1	9

immune-mediated diseases										

Appendix S6: Risk of bias results.

Four of the 17 included randomized controlled trials had unclear risk of bias from inadequate reporting of random sequence generation (23%) and 5 from inadequate reporting of allocation concealment (29%). In addition, 8 studies had a high risk of "other" biases
(47%).

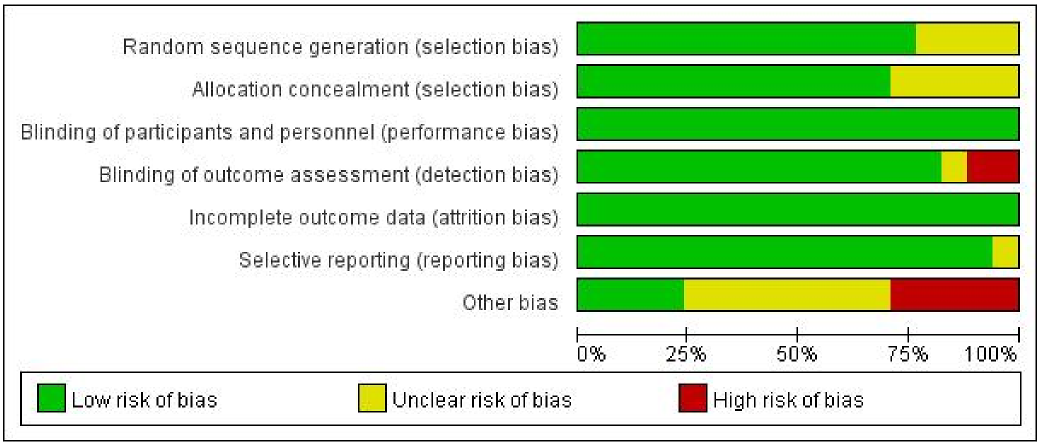

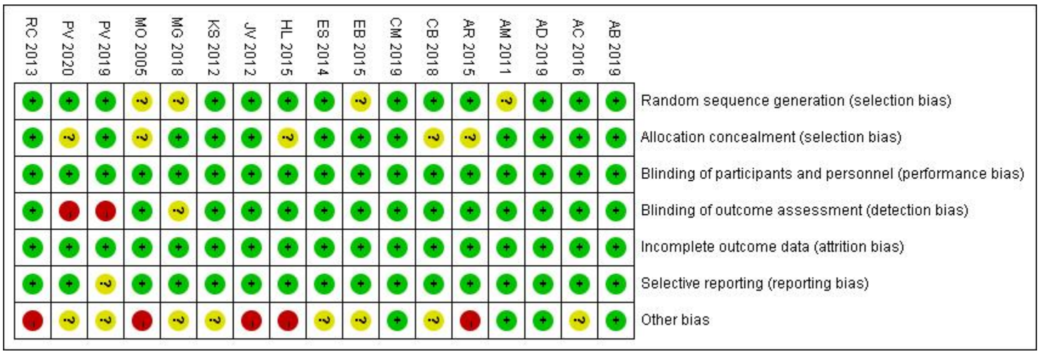

Appendix S7 :Three comparators were included: live attenuated vaccine, adjuvant recombinant subunit vaccine, and placebo.

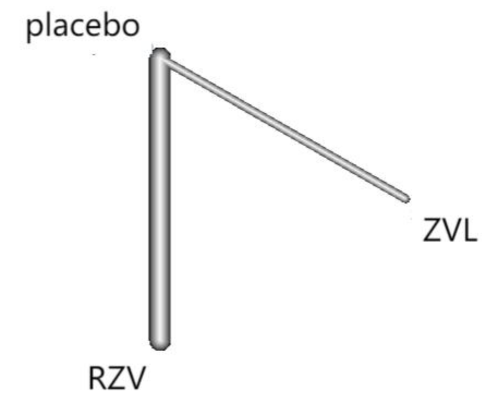

Appendix S8: The funnel plots and publication bias plots.

RZV

Injection sites

Redness:

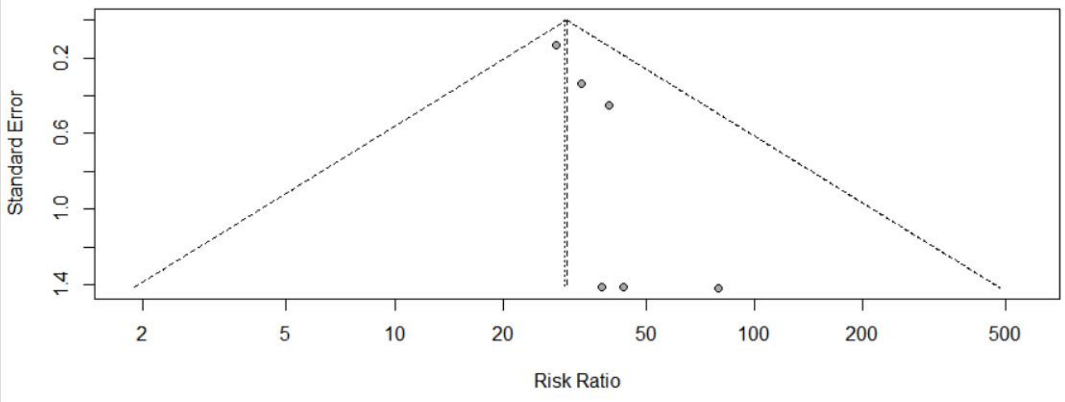

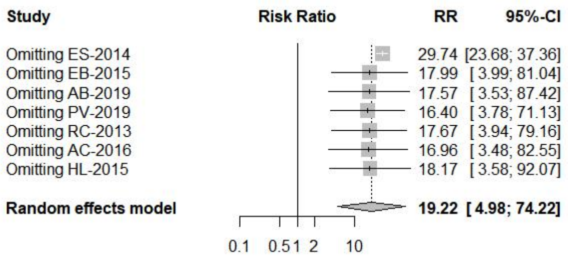

Swelling:

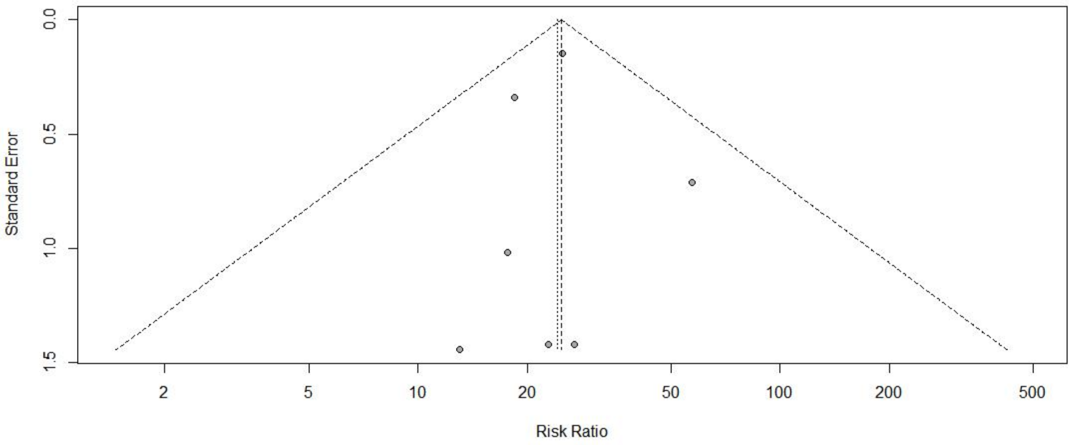

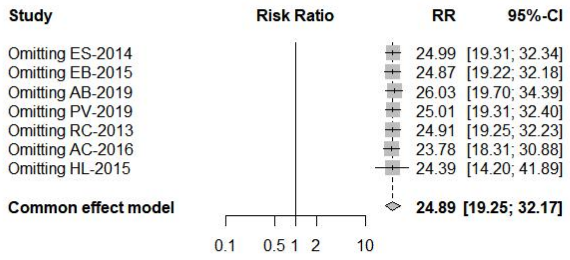

Pain:

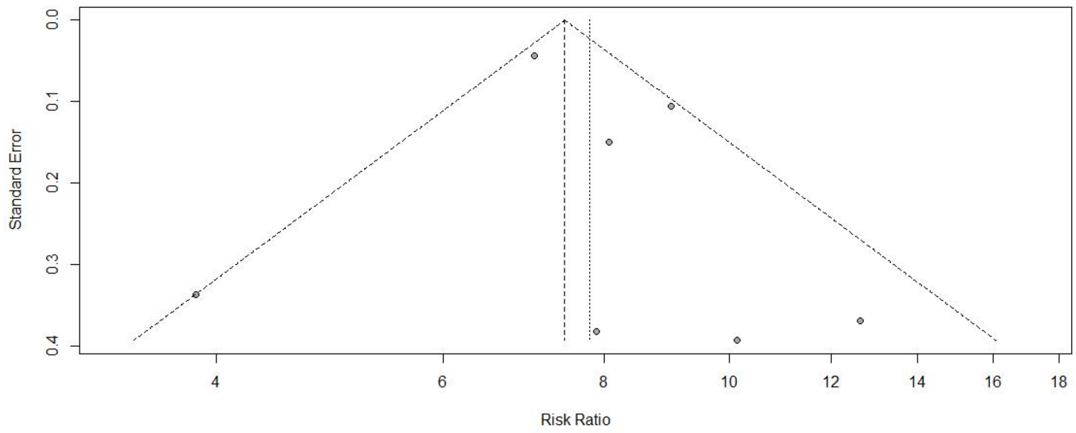

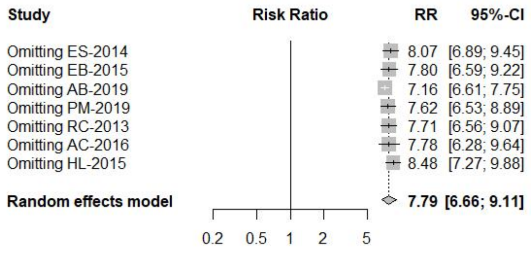

Systemic adverse events

Fatigue：

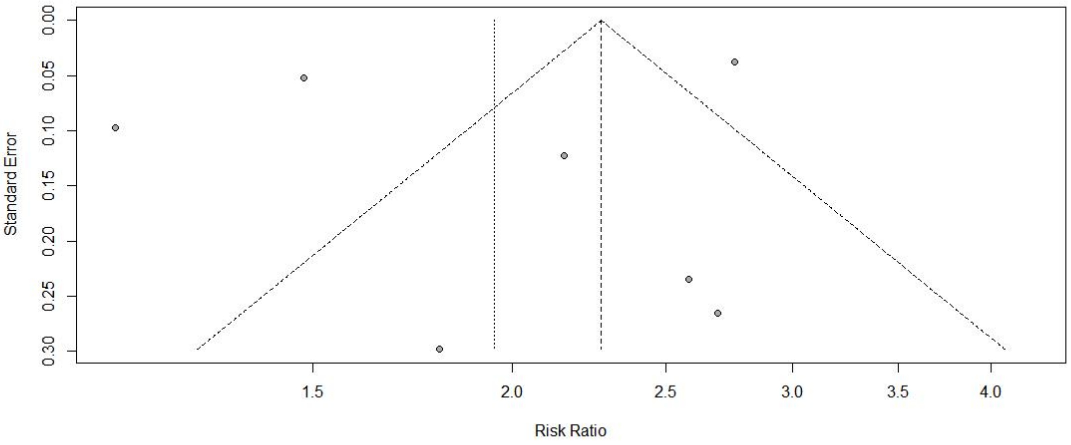
Standard Error
0.00
0.05
0.10
0.15
0.20
0.25
0.30
1.5
2.0
2.5
3.0
3.5
4.0
Risk Ratio

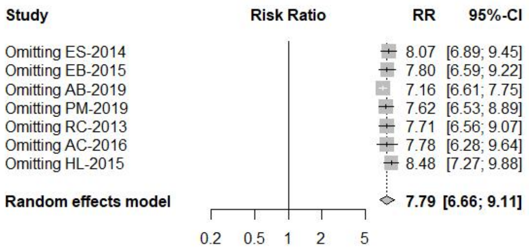
Study
Risk Ratio
RR
95%-CI
Omitting ES-2014 8.07 [6.89; 9.45]
Omitting EB-2015 7.80 [6.59; 9.22]
Omitting AB-2019 7.16 [6.61; 7.75]
Omitting PM-2019 7.62 [6.53; 8.89]
Omitting RC-2013 7.71 [6.56; 9.07]
Omitting AC-2016 7.78 [6.28; 9.64]
Omitting HL-2015 8.48 [7.27; 9.88]
Random effects model 7.79 [6.66; 9.11]
0.2
0.5
1
2
5

Myalgia：

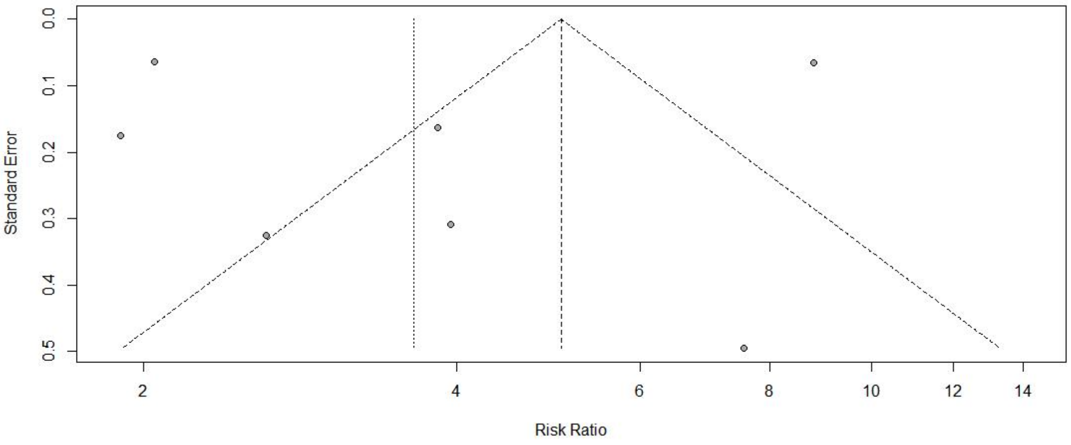
Standard Error
0.0
0.1
0.2
0.3
0.4
0.5
2
4
6
8
10
12
14
Risk Ratio

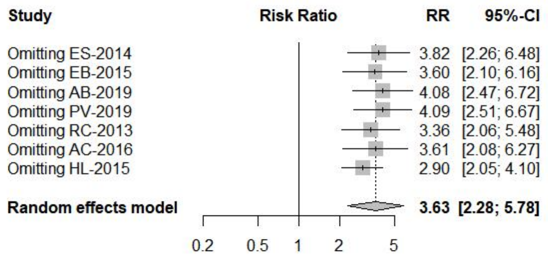

Gastrointestinal symptoms:

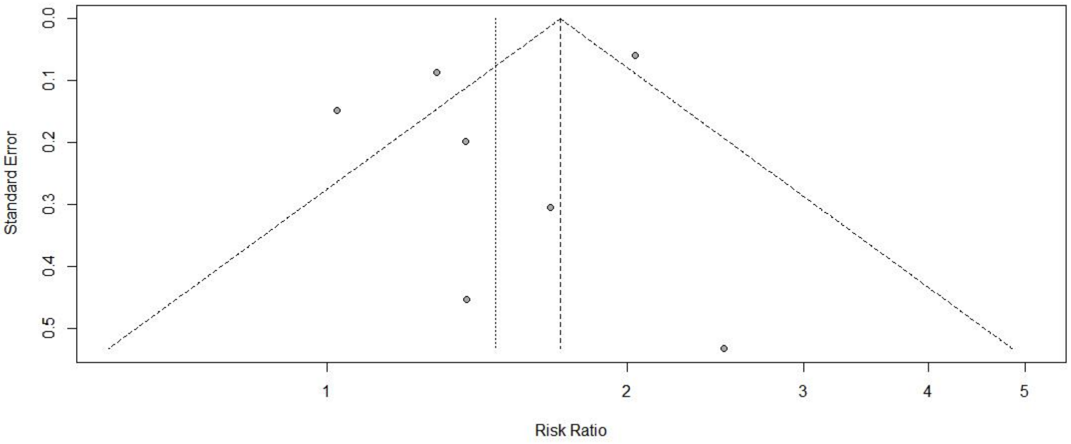

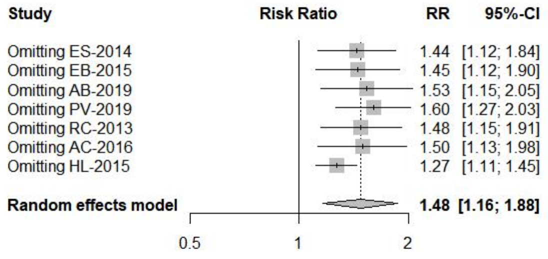

Headache：

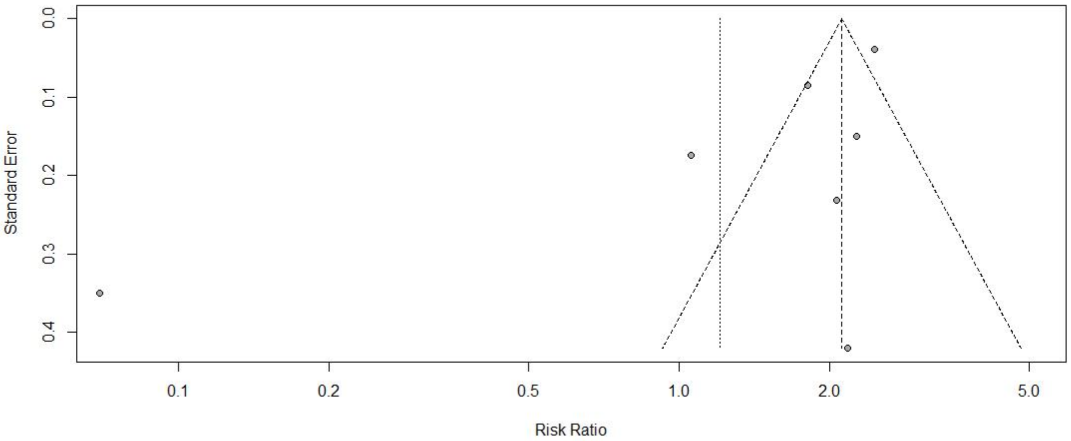

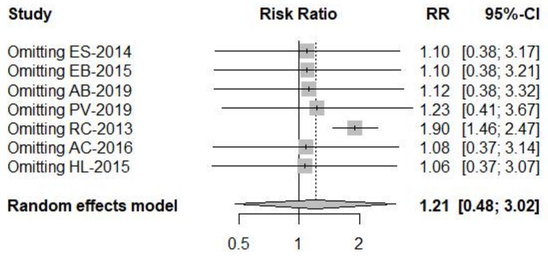

Serious adverse events

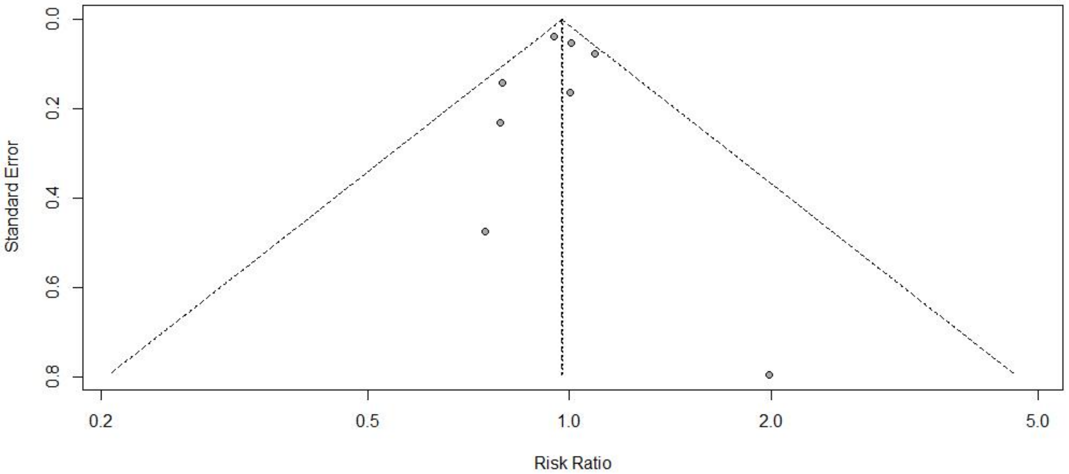

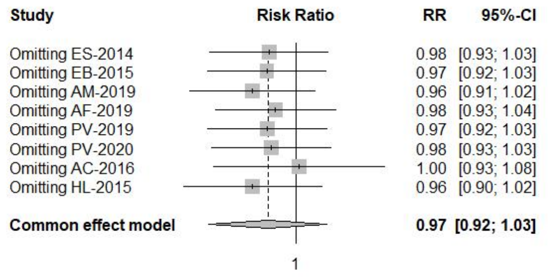

Death

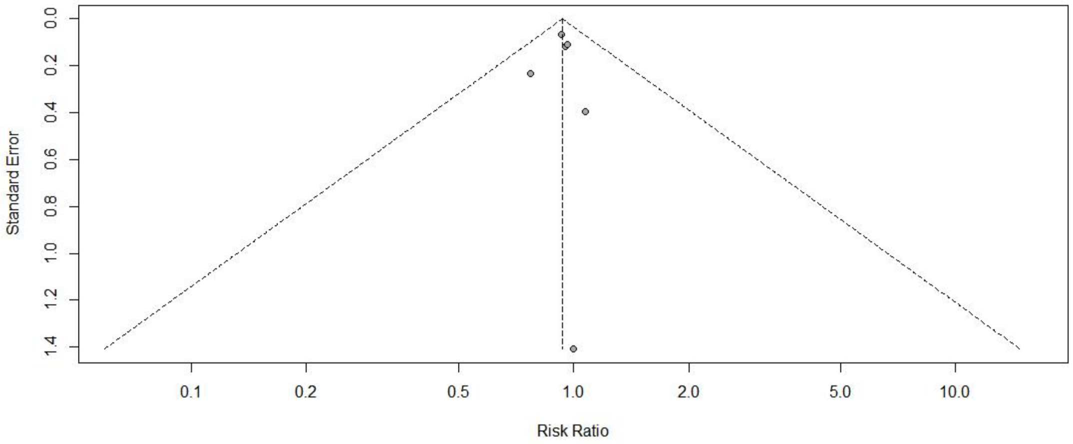

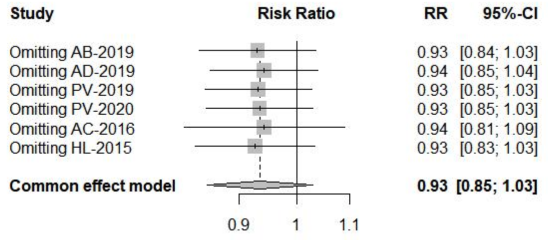

ZVL
Injection sites

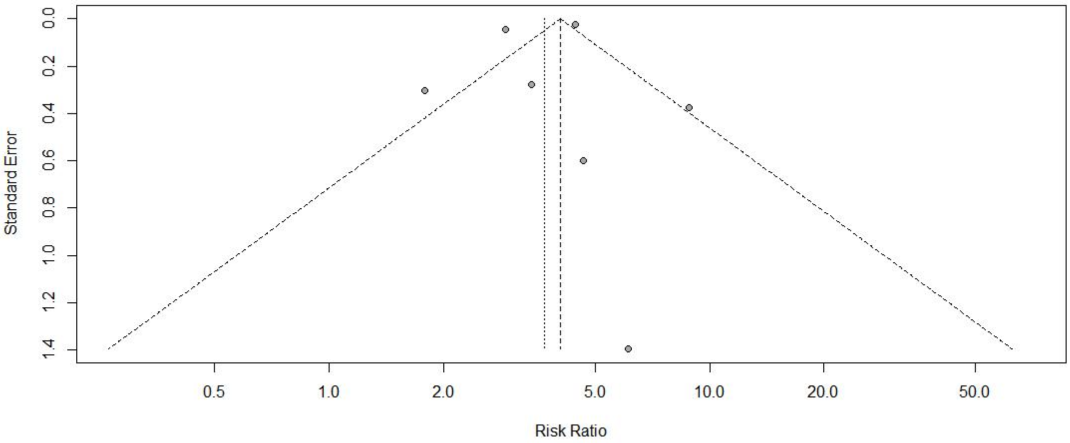

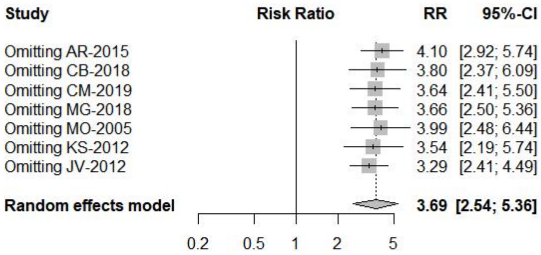

Systemic adverse events

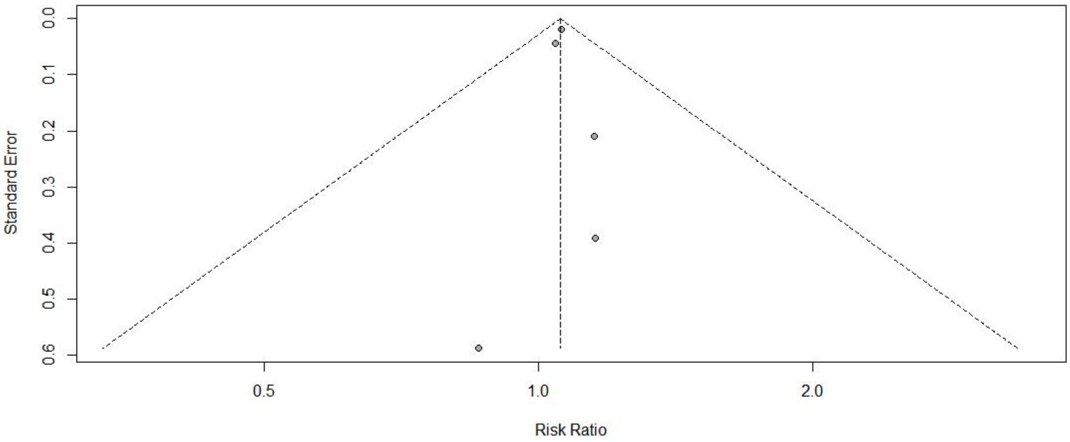

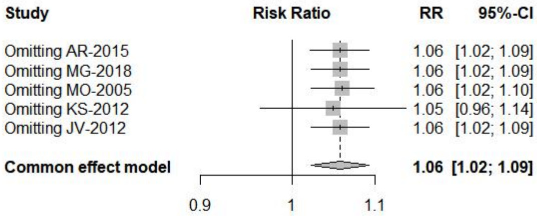

Serious adverse events

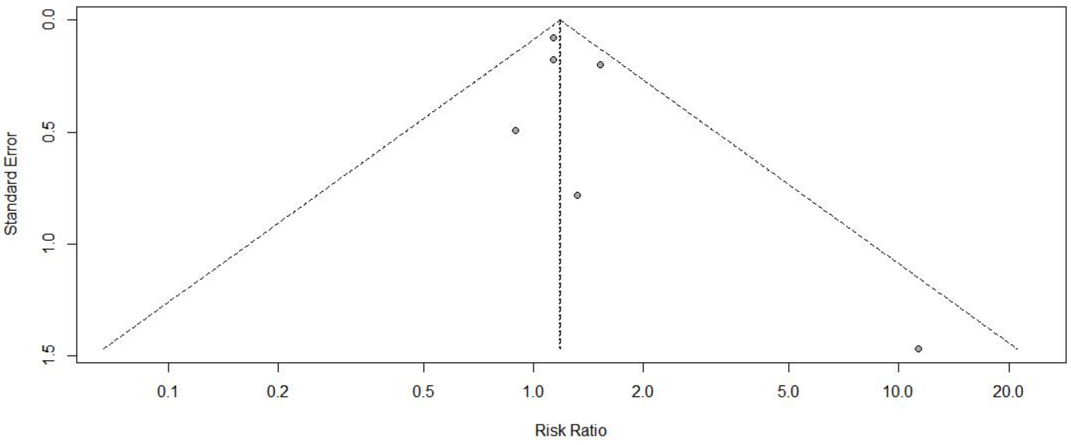

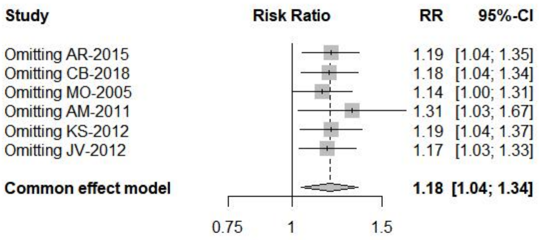

Death

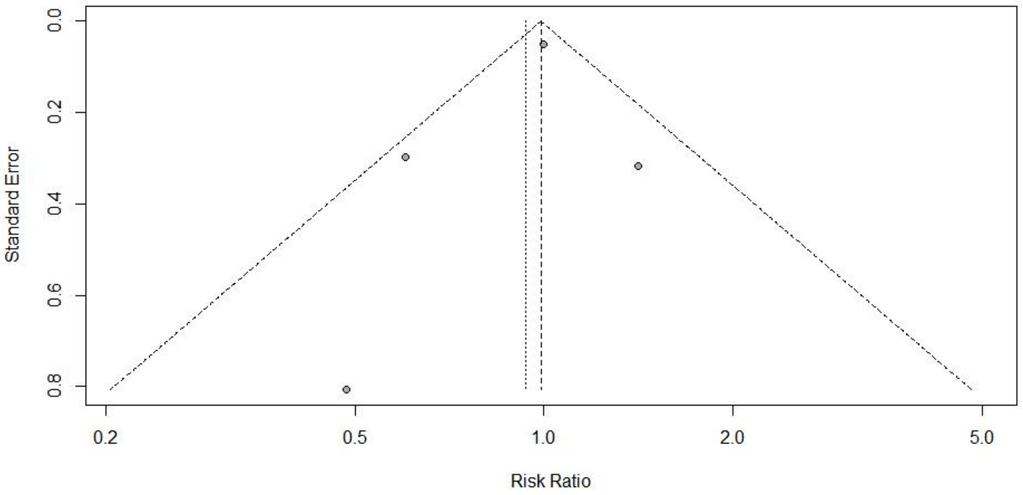

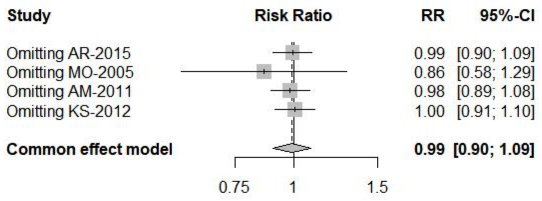
Study
Risk Ratio
RR
95%-CI
Omitting AR-2015
0.99 [0.90; 1.09]
Omitting MO-2005
0.86 [0.58; 1.29]
Omitting AM-2011
0.98 [0.89; 1.08]
Omitting KS-2012
1.00 [0.91; 1.10]
Common effect model
0.99 [0.90; 1.09]
0.75
1
1.5

Appendix S9: Forest plot of RZV vaccine efficacy by age subgroup.

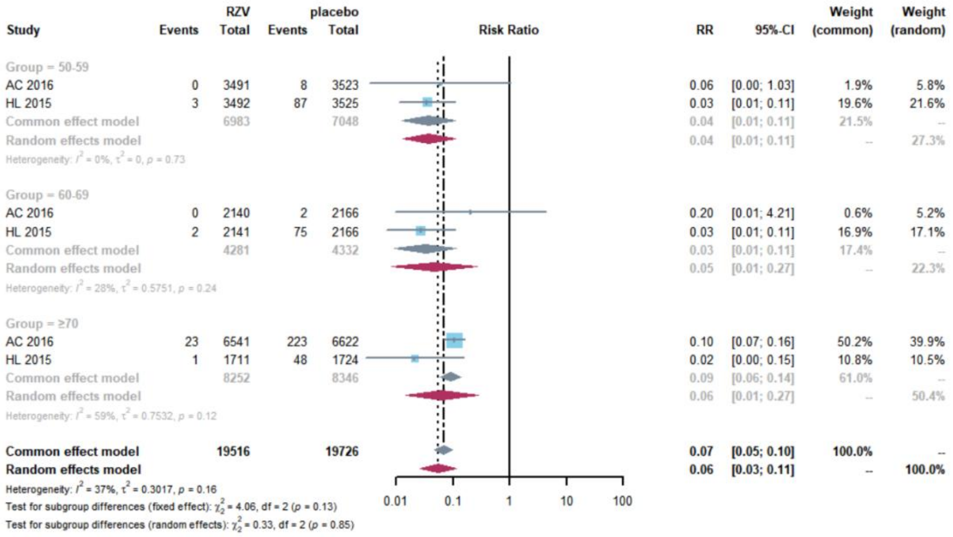

Appendix S10: Forest plot of ZVL vaccine effectiveness by age subgroup.

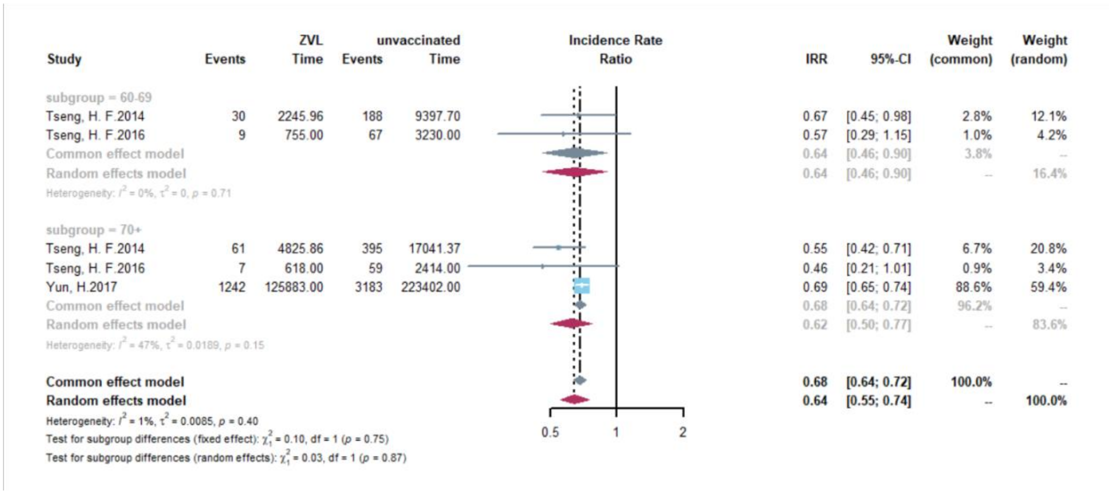

Appendix S11: Forest plot of RZV vaccine effectiveness for prevention of HZO.

Study	RZV Events	RZV Time	unvaccinated Events	unvaccinated Time	IRR	95%-CI	Weight (common)	Weight (random)
Izurieta, H. S.2021	157	618	19306	25235	0.33	[0.28; 0.39]	83.0%	83.5%
Lu, A.2021	30	117517	5654	7374053	0.33	[0.23; 0.48]	16.0%	16.0%
Sun, Y.2021	1	8404	87	120739	0.17	[0.02; 1.19]	1.0%	0.5%
**Common effect model**					**0.33**	**[0.29; 0.38]**	**100.0%**	--
**Random effects model**					**0.33**	**[0.29; 0.38]**	--	**100.0%**

Heterogeneity: $I^2 = 0\%$, $\tau^2 = 0$, $p = 0.79$

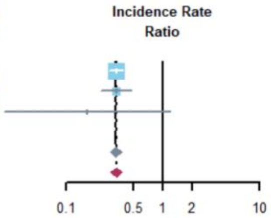

Appendix S12: Safety outcomes of RZV.

Injection sites

Redness:

Study	RZV Events	RZV Total	placebo Events	placebo Total	RR	95%-CI	Weight (common)	Weight (random)
Group = immunocompromised								
EB 2015	28	73	0	48	37.61	[2.35; 601.69]	0.8%	0.7%
AB 2019	301	901	9	892	33.11	[17.17; 63.84]	12.1%	12.1%
PV 2019	40	112	0	110	79.56	[4.95; 1277.93]	0.7%	0.7%
Common effect model		1086		1050	35.69	[19.14; 66.57]	13.6%	--
Random effects model					34.83	[18.69; 64.91]	--	13.4%
Heterogeneity: $I^2 = 0\%$, $\tau^2 = 0$, $p = 0.83$								
Group = immunocompetent								
RC 2013	44	150	0	73	43.47	[2.71; 695.97]	0.9%	0.7%
AC 2016	198	505	5	505	39.60	[16.44; 95.37]	6.7%	6.7%
HL 2015	1664	4382	59	4377	28.17	[21.80; 36.40]	78.9%	79.2%
Common effect model		5037		4955	29.21	[22.86; 37.34]	86.4%	--
Random effects model					29.02	[22.72; 37.08]	--	86.6%
Heterogeneity: $I^2 = 0\%$, $\tau^2 = 0$, $p = 0.74$								
**Common effect model**		**6123**		**6005**	**30.09**	**[23.95; 37.81]**	**100.0%**	--
**Random effects model**					**29.74**	**[23.68; 37.36]**	--	**100.0%**

Heterogeneity: $I^2 = 0\%$, $\tau^2 = 0$, $p = 0.94$
Test for subgroup differences (fixed effect): $\chi^2_1 = 0.34$, df = 1 ($p = 0.56$)
Test for subgroup differences (random effects): $\chi^2_1 = 0.28$, df = 1 ($p = 0.59$)

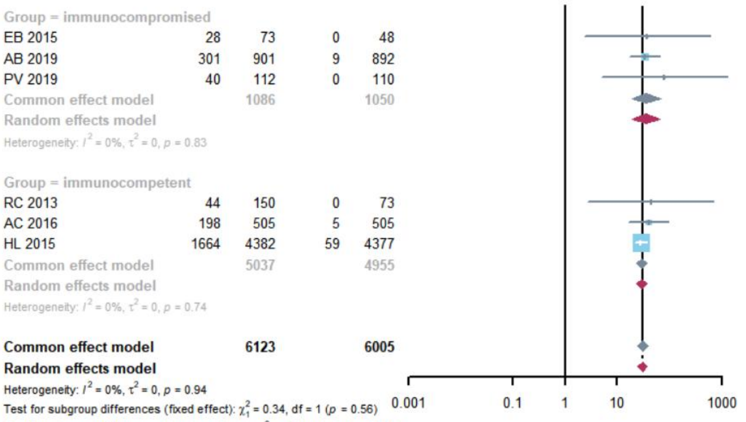

Swelling:

Study	RZV Events	RZV Total	placebo Events	placebo Total	RR	95%-CI	Weight (common)	Weight (random)
Group = immunocompromised								
ES 2014	6	30	0	30	13.00	[0.77; 220.80]	0.8%	0.8%
EB 2015	20	73	0	48	27.05	[1.68; 436.91]	1.0%	0.9%
AB 2019	168	901	9	892	18.48	[9.51; 35.90]	15.1%	14.9%
PV 2019	18	112	1	110	17.68	[2.40; 130.16]	1.7%	1.7%
Common effect model		1116		1080	18.63	[10.20; 34.02]	18.6%	--
Random effects model					18.44	[10.11; 33.63]	--	18.3%
Heterogeneity: $I^2 = 0\%$, $\tau^2 = 0$, $p = 0.99$								
Group = immunocompetent								
RC 2013	23	150	0	73	22.95	[1.41; 372.67]	1.1%	0.8%
AC 2016	114	505	2	505	57.00	[14.16; 229.43]	3.3%	3.4%
HL 2015	1153	4382	46	4377	25.04	[18.70; 33.52]	76.9%	77.5%
Common effect model		5037		4955	26.32	[19.81; 34.97]	81.4%	--
Random effects model					25.88	[19.48; 34.39]	--	81.7%
Heterogeneity: $I^2 = 0\%$, $\tau^2 = 0$, $p = 0.52$								
**Common effect model**		**6153**		**6035**	**24.89**	**[19.25; 32.17]**	**100.0%**	--
**Random effects model**					**24.33**	**[18.82; 31.45]**	--	**100.0%**

Heterogeneity: $I^2 = 0\%$, $\tau^2 = 0$, $p = 0.88$
Test for subgroup differences (fixed effect): $\chi^2_1 = 1.04$, df = 1 ($p = 0.31$)
Test for subgroup differences (random effects): $\chi^2_1 = 1.00$, df = 1 ($p = 0.32$)

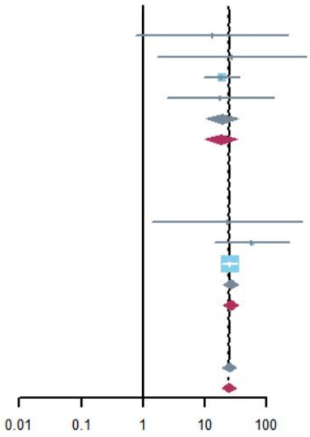

Pain:

Study	RZV Events	RZV Total	placebo Events	placebo Total	RR	95%-CI	Weight (common)	Weight (random)
Group = immunocompromised								
ES 2014	27	30	7	30	3.86	[1.99; 7.46]	1.1%	5.0%
EB 2015	72	73	6	48	7.89	[3.73; 16.69]	1.1%	4.0%
AB 2019	756	901	83	892	9.02	[7.33; 11.09]	12.9%	25.3%
PM 2019	90	112	7	110	12.63	[6.13; 26.01]	1.1%	4.3%
Common effect model		1116		1080	8.84	[7.34; 10.64]	16.2%	--
Random effects model					7.81	[5.05; 12.09]	--	38.6%
Heterogeneity: $I^2 = 57\%$, $\tau^2 = 0.1148$, $p = 0.07$								
Group = immunocompetent								
RC 2013	125	150	6	73	10.14	[4.69; 21.90]	1.2%	3.8%
AC 2016	347	505	43	505	8.07	[6.03; 10.80]	6.7%	17.6%
HL 2015	3464	4382	490	4377	7.06	[6.49; 7.69]	75.9%	40.0%
Common effect model		5037		4955	7.19	[6.63; 7.79]	83.8%	--
Random effects model					7.16	[6.61; 7.77]	--	61.4%
Heterogeneity: $I^2 = 0\%$, $\tau^2 = 0$, $p = 0.46$								
**Common effect model**		**6153**		**6035**	**7.45**	**[6.92; 8.03]**	**100.0%**	--
**Random effects model**					**7.79**	**[6.66; 9.11]**	--	**100.0%**

Heterogeneity: $I^2 = 48\%$, $\tau^2 = 0.0141$, $p = 0.07$
Test for subgroup differences (fixed effect): $\chi^2_1 = 4.01$, df = 1 ($p = 0.05$)
Test for subgroup differences (random effects): $\chi^2_1 = 0.15$, df = 1 ($p = 0.70$)

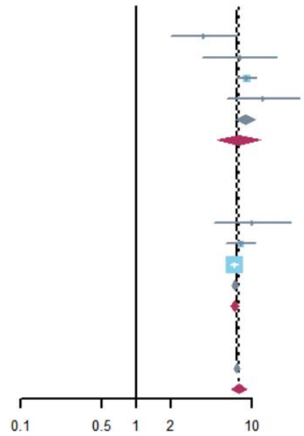

Systemic adverse events

Fatigue:

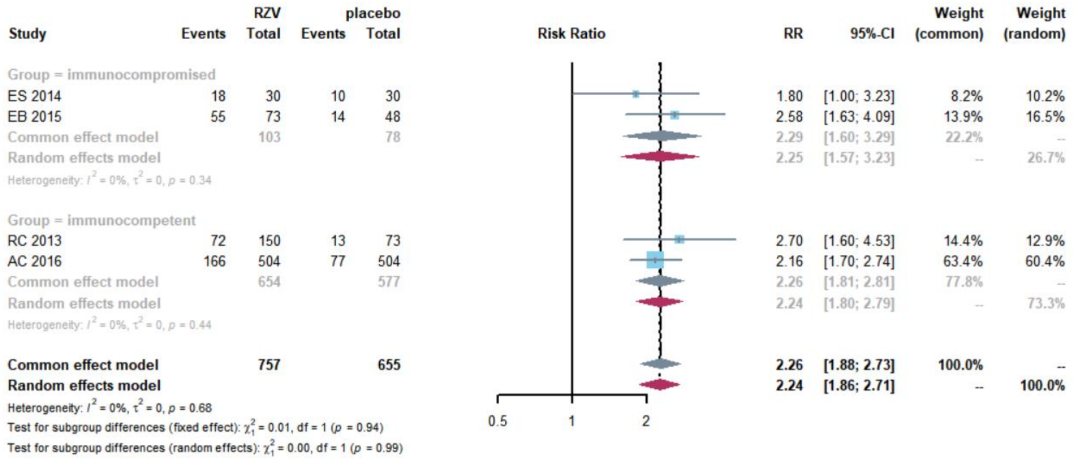

Myalgia：

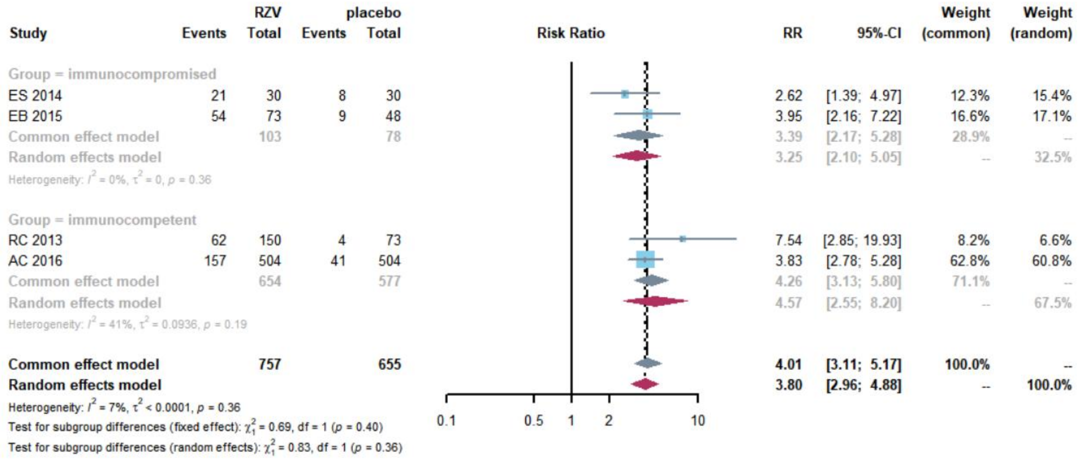

Headache：

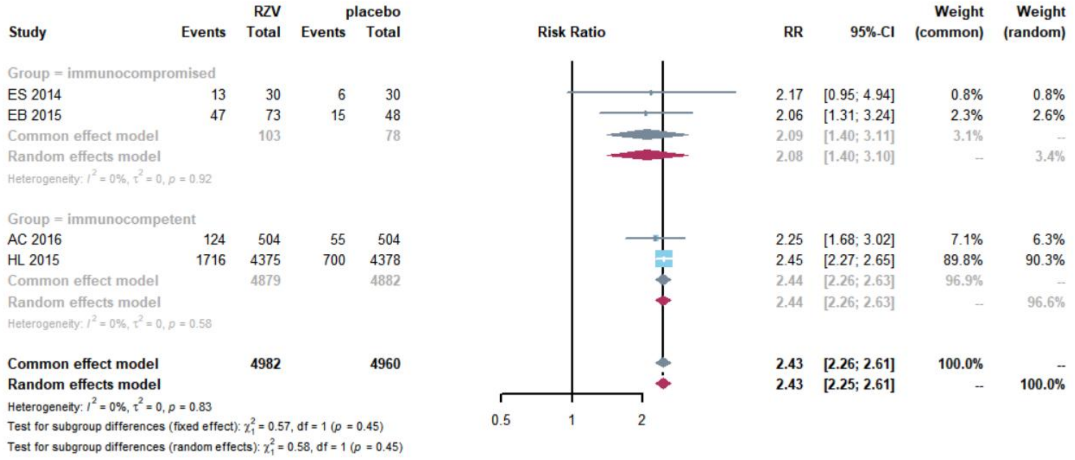

Gastrointestinal symptoms:

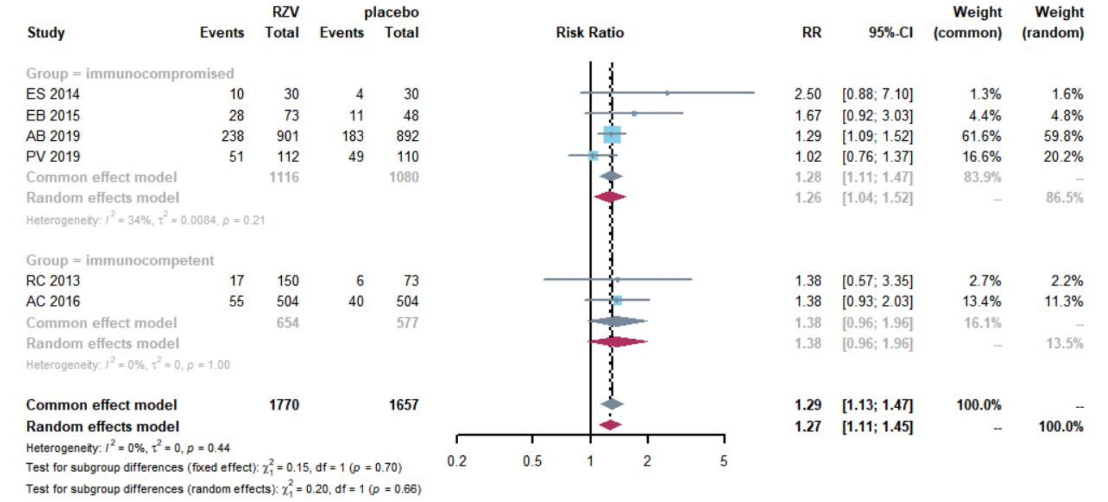

Serious adverse events

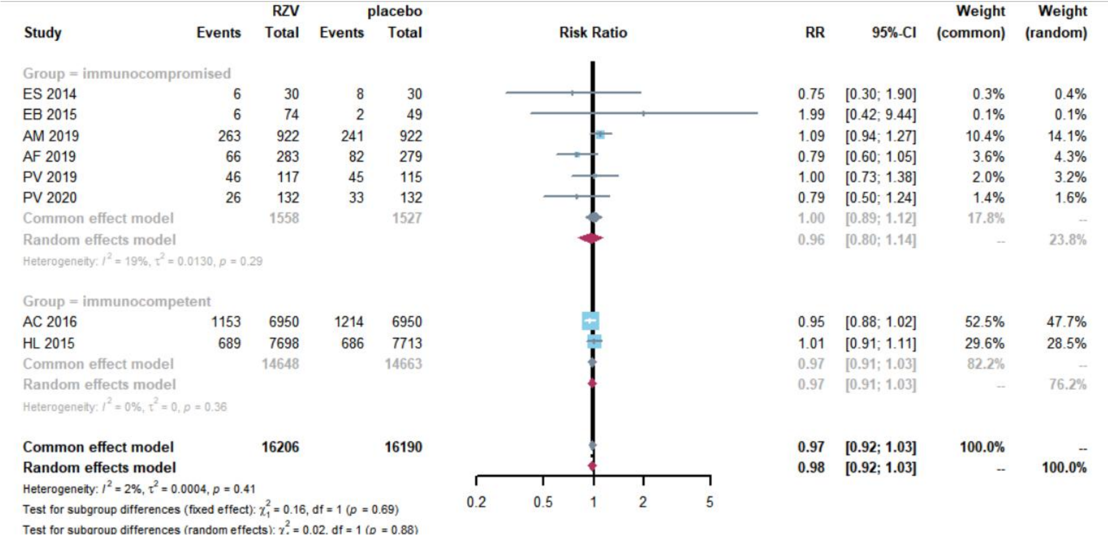

Death

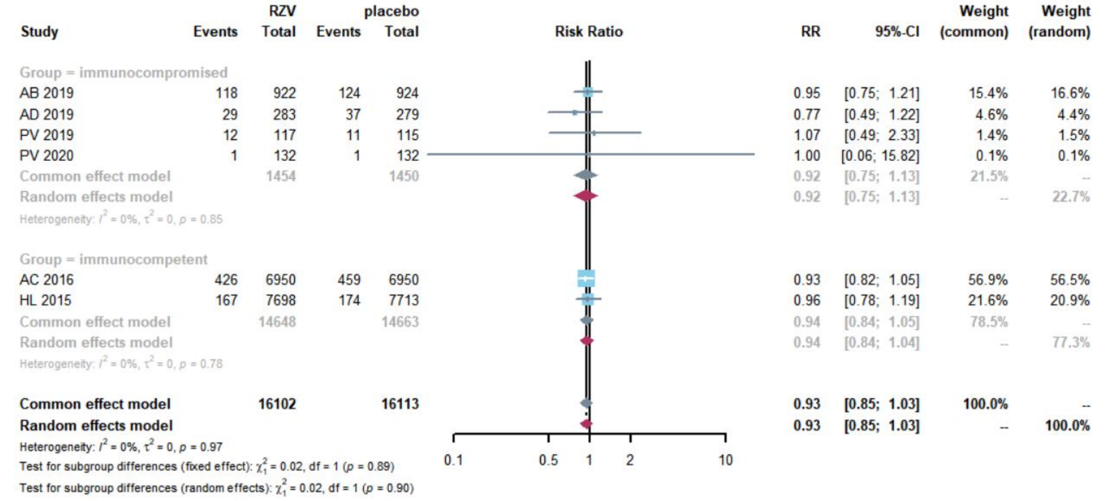

Appendix S13：Safety outcomes of ZVL.

Injection sites

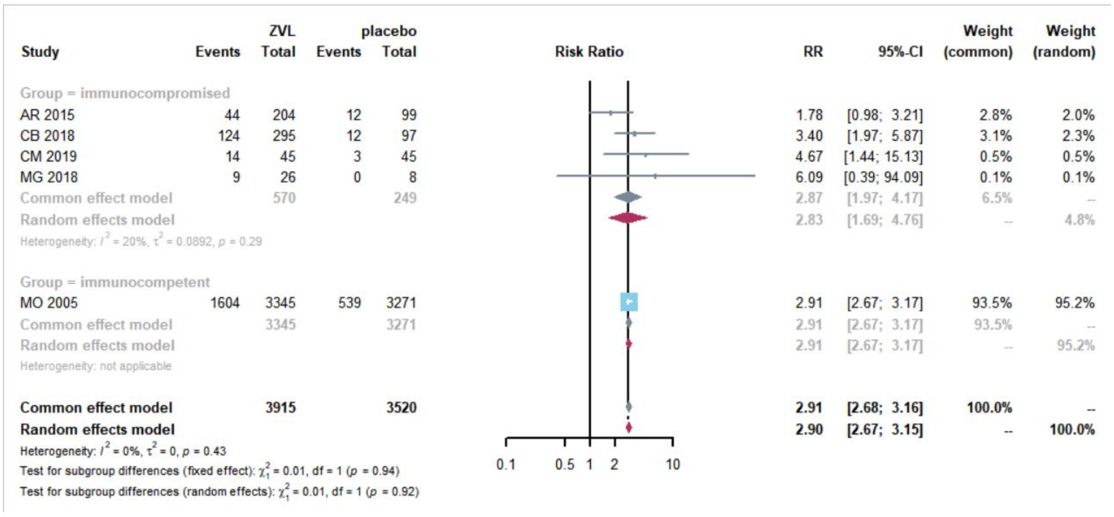

Systemic adverse events

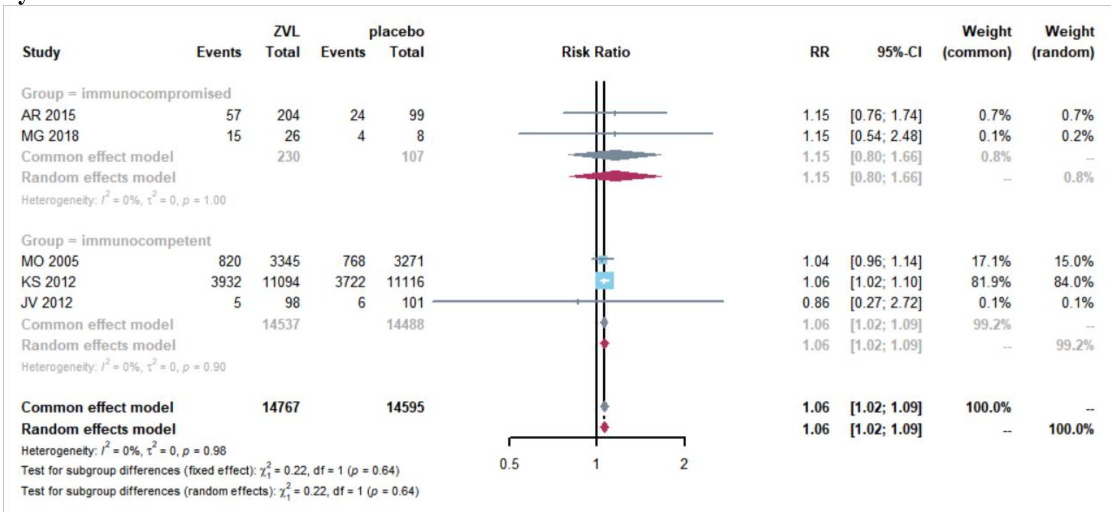

Serious adverse events

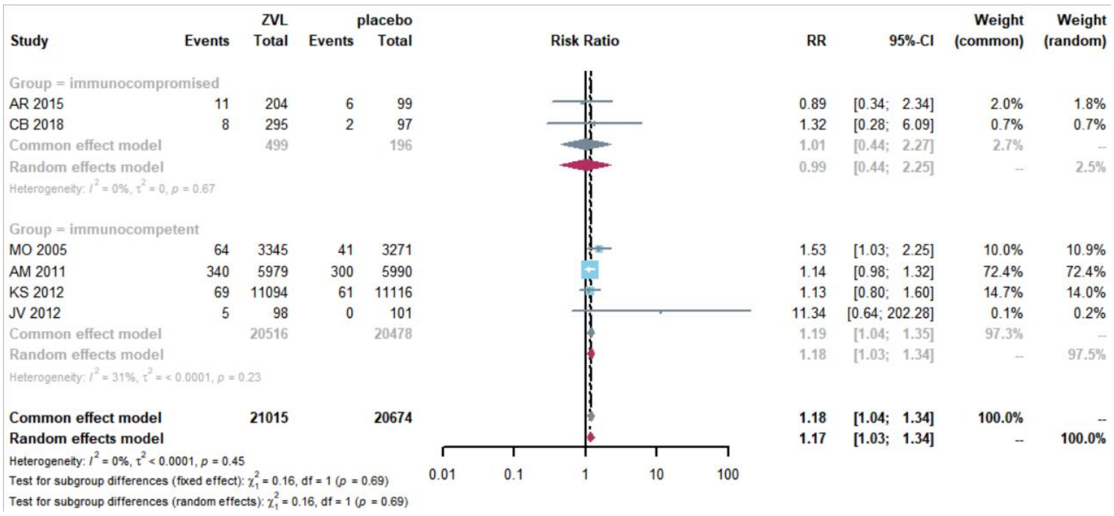

Death

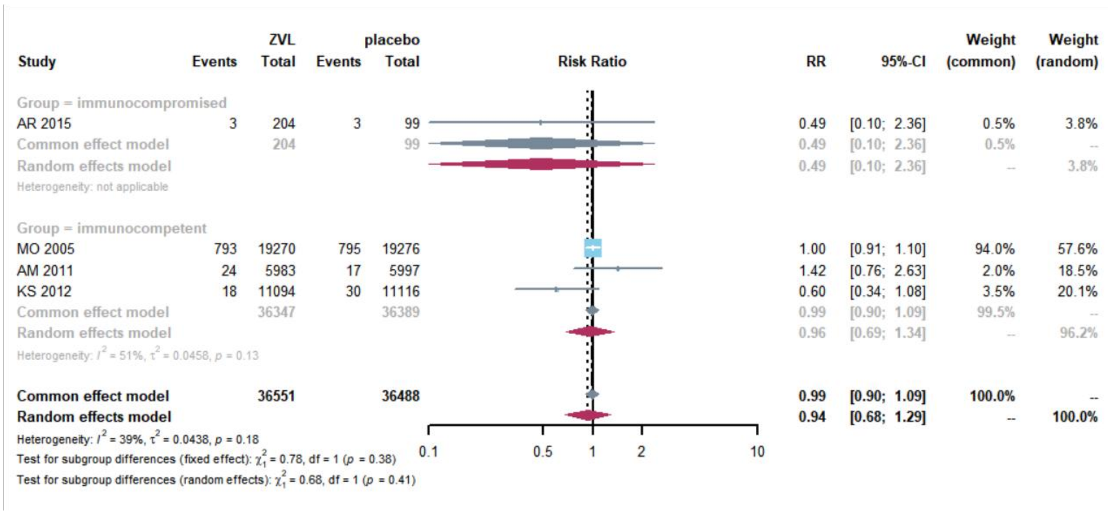
ZVL
placebo
Study
Events
Total
Events
Total
Risk Ratio
RR
95%-CI
Weight (common)
Weight (random)
Group = immunocompromised
AR 2015 3 204 3 99 0.49 [0.10; 2.36] 0.5% 3.8%
Common effect model 204 99 0.49 [0.10; 2.36] 0.5% --
Random effects model 0.49 [0.10; 2.36] -- 3.8%
Heterogeneity: not applicable
Group = immunocompetent
MO 2005 793 19270 795 19276 1.00 [0.91; 1.10] 94.0% 57.6%
AM 2011 24 5983 17 5997 1.42 [0.76; 2.63] 2.0% 18.5%
KS 2012 18 11094 30 11116 0.60 [0.34; 1.08] 3.5% 20.1%
Common effect model 36347 36389 0.99 [0.90; 1.09] 99.5% --
Random effects model 0.96 [0.69; 1.34] -- 96.2%
Heterogeneity: I^2 = 51%, τ^2 = 0.0458, p = 0.13
Common effect model 36551 36488 0.99 [0.90; 1.09] 100.0% --
Random effects model 0.94 [0.68; 1.29] -- 100.0%
Heterogeneity: I^2 = 39%, τ^2 = 0.0438, p = 0.18
Test for subgroup differences (fixed effect): χ^2_1 = 0.78, df = 1 (p = 0.38)
Test for subgroup differences (random effects): χ^2_1 = 0.68, df = 1 (p = 0.41)
0.1
0.5
1
2
10
